# Identification and functional characterization of a flax UDP-glycosyltransferase glucosylating secoisolariciresinol (SECO) into secoisolariciresinol monoglucoside (SMG) and diglucoside (SDG)

**DOI:** 10.1186/1471-2229-14-82

**Published:** 2014-03-28

**Authors:** Kaushik Ghose, Kumarakurubaran Selvaraj, Jason McCallum, Chris W Kirby, Marva Sweeney-Nixon, Sylvie J Cloutier, Michael Deyholos, Raju Datla, Bourlaye Fofana

**Affiliations:** 1 Crops and Livestock Research Centre, Agriculture and Agri-Food Canada, 440 University Avenue, Charlottetown, PE C1A 4 N6, Canada; 2 Department of Biology, University of Prince Edward Island, 550 University Avenue, Charlottetown, PE C1A 4P3, Canada; 3 Cereal Research Centre, Agriculture and Agri-Food Canada, 195 Dafoe Road, Winnipeg, MB R3T 2 M9, Canada; 4 Department of Biological Sciences, University of Alberta, Edmonton, AB T6G 2E9, Canada; 5 National Research Council, 110 Gymnasium Place, Saskatoon, SK S7N 0 W9, Canada

**Keywords:** Flax, Lignan, UGTs, SDG, Secoisolariciresinol, Glucosylation, Glycosyltranferases

## Abstract

**Background:**

Lignans are a class of diphenolic nonsteroidal phytoestrogens often found glycosylated *in planta*. Flax seeds are a rich source of secoisolariciresinol diglucoside (SDG) lignans. Glycosylation is a process by which a glycosyl group is covalently attached to an aglycone substrate and is catalyzed by uridine diphosphate glycosyltransferases (UGTs). Until now, very little information was available on UGT genes that may play a role in flax SDG biosynthesis. Here we report on the identification, structural and functional characterization of 5 putative UGTs potentially involved in secoisolariciresinol (SECO) glucosylation in flax.

**Results:**

Five UGT genes belonging to the glycosyltransferases’ family 1 (EC 2.4.x.y) were cloned and characterized. They fall under four UGT families corresponding to five sub-families referred to as *UGT74S1*, *UGT74T1*, *UGT89B3*, *UGT94H1*, *UGT712B1* that all display the characteristic plant secondary product glycosyltransferase (PSPG) conserved motif. However, diversity was observed within this 44 amino acid sequence, especially in the two peptide sequences WAPQV and HCGWNS known to play a key role in the recognition and binding of diverse aglycone substrates and in the sugar donor specificity. In developing flax seeds, *UGT74S1* and *UGT94H1* showed a coordinated gene expression with that of pinoresinol-lariciresinol reductase (PLR) and their gene expression patterns correlated with SDG biosynthesis. Enzyme assays of the five heterologously expressed UGTs identified UGT74S1 as the only one using SECO as substrate, forming SECO monoglucoside (SMG) and then SDG in a sequential manner.

**Conclusion:**

We have cloned and characterized five flax UGTs and provided evidence that UGT74S1 uses SECO as substrate to form SDG *in vitro*. This study allowed us to propose a model for the missing step in SDG lignan biosynthesis.

## Background

Lignans are a class of diphenolic nonsteroidal phytoestrogens with a wide variety of purported health benefits [1-4]. Different types of lignans have been reported in various plant species and include secoisolariciresinol diglucoside (SDG) found mainly in flax (*Linum usitatissimum* L.) [5-10]. Flax seeds are a rich source of SDG lignans that have been associated with positive roles in the prevention of chronic metabolic diseases in human [11-14].

*In planta,* lignans are usually found glycosylated in oligomeric chains [15]. Glycosylation is a key mechanism that determines the chemical complexity and diversity of plant natural products [16,17], ensures their chemical stability and water solubility while reducing chemical reactivity or toxicity [18], and facilitates their sorting, intercellular transport, storage and accumulation in plant cells [19,20]. Glycosylation is one of the key modifications in the secondary metabolite biosynthesis and is catalyzed by carbohydrate active enzymes (CAZymes) which include the superfamily of glycosyltransferases (GTs) [21]. The specific glycosylation position in biologically active compounds may serve to modulate their pharmacological activity and/or to alter and optimize their potential use as drugs [17]. Members of the GT superfamily have been classified into 94 families where family 1 refers to the uridine glycosyl transferases (UGTs) [22,23]. Plant UGTs are often characterized by a 44 amino acid consensus signature motif, the plant secondary product glycosyltransferase (PSPG) box [15,23,24]. UGTs transfer UDP-activated sugar moieties, including UDP-glucose, to specific acceptor molecules [25]. Based on sequence homology, more than 120 UGTs have been reported in *Arabidopsis* and were grouped into 30 sub-families classified as UGT71 to UGT100 [22]. In the course of this study, the flax draft genome was released [26]. Barvkar et al. [27] probed this flax draft genome and reported 137 flax UGTs but did not assign functions to any of these UGTs.

**Figure 1:**
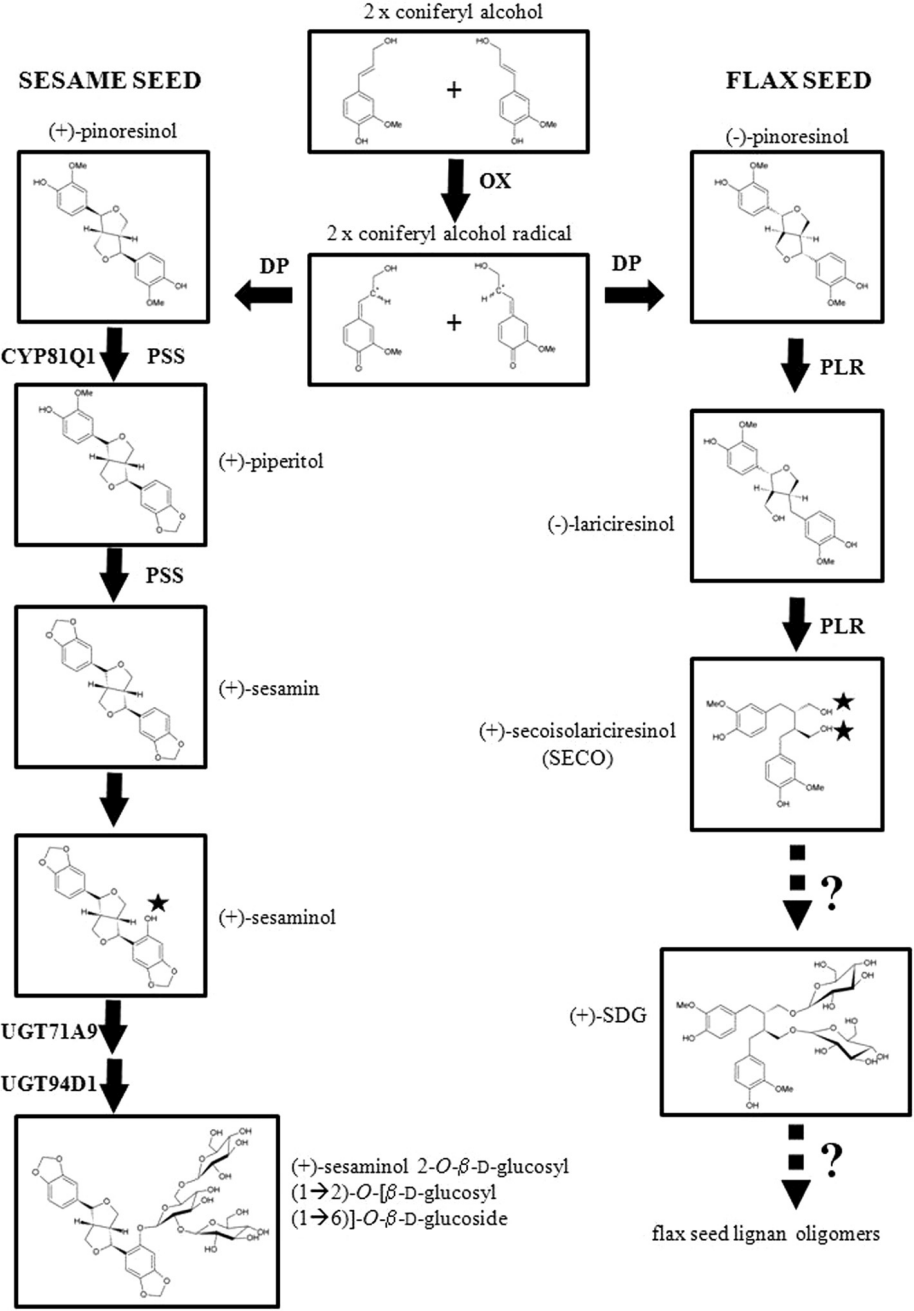
**Lignan biosynthesis pathways in sesame and flax starting from coniferyl alcohol.** OX, oxidation, DP, dirigent protein; PLR, pinoresinol-lariciresinol reductase; PSS, piperitol sesamin synthase; SDG, secoisoalariciresinol diglucoside. Stars indicate the hydroxyl groups glycosylated in sesaminol and secoisolariciresinol. Adapted from Kim et al. [31] with the permission of Dr. Honoo Satake and the PCP editorial office.

Pinoresinol-lariciresinol reductases (PLRs) are key enzymes for the catalysis of the first biosynthetic steps of lignans in many plant species, including flax. These enzymes sequentially reduce pinoresinol formed by the coupling of two molecules of coniferyl alcohol (Figure 1) in the presence of dirigent proteins [28]. Recently, Noguchi et al. [6] reported two UGTs, UGT71A9 and UGT94D1, that sequentially glycosylated furofuran lignan (+)-sesaminol in *Sesamum indicum* to form (+)-sesaminol 2–O–β-D-glucosyl (1–2)-O-[β-D-glucosyl(1–6)]-β-D-glucoside (STG)*.* STG and SDG are structurally quite different. In STG, the glucosyl moieties form a trisaccharide side chain while in SDG, the sugars are attached at two different hydroxyl groups of the secoisolariciresinol backbone (Figure 1). Hence, the UGTs that glycosylate sesamine into sesaminol are likely to differ from those glycosylating secoisolaricresinol (SECO). Although cDNAs encoding for PLRs that specifically convert pinoresinol into (−) and (+) enantiomers of SECO have been cloned and functionally characterized in flax [28-31], much less is known about the UGTs that glucosylate SECO aglycones into SDG in flax.

To gain insights into SDG lignan glucosylation with potential applications in lignan metabolism engineering, we attempted to identify and characterize flax UGTs responsible for SECO glucosylation. Using database mining, molecular cloning, heterologous expression and enzyme assays, we isolated five putative UDP-glycosyltransferases from flax seeds and demonstrated that UGT74S1 glucosylated SECO, forming sequentially SECO monoglucoside (SMG) and then SDG. The findings, not only reported the first functional characterization of a SECO specific UGT in flax, but also pave the way for engineered SDG lignan metabolite species *in vitro* and *in planta*.

## Results

### Library mining and UGT cloning

Using 19 NAPGEN EST library-derived gene-specific UGT primers and one degenerate (UGT-F2) primer, a total of 16 combinations produced unique PCR products of the expected sizes. The partial cDNA sequences were analyzed using BLASTx which confirmed the identity of each sequence as belonging to the UGT family. A ClustalW multiple sequence alignment showed that some of them were the same and a consensus phylogenetic tree revealed that eight were unique (Additional file 1). Subsequently, one representative sequence from each of the eight UGTs was selected for the design of gene-specific primers, and full length cDNAs for five different UGTs were obtained (Additional file 2A-C). *CL5227* was 1.2 kb while *CL809*, *CL8584*, *RP131*, and *RP250* were all ~1.5 kb (Additional file 2C). The unique UGT sequences were classified as belonging to four families and five sub-families as per the nomenclature of the International Union of Biochemistry and Molecular Biology and the IUPAC-IUBMB joint committee responsible for UDP-glycosyltransferases [32] and designated *UGT74S1* (*CL809*), *UGT94H1* (*CL5227*), *UGT89B3* (*CL8584*), *UGT74T1* (*RP131*) and *UGT712B1* (*RP250*). Their sequences were submitted to GenBank under accession numbers JX011632 to JX011636.

### UGT structural gene organization

**Figure 2:**
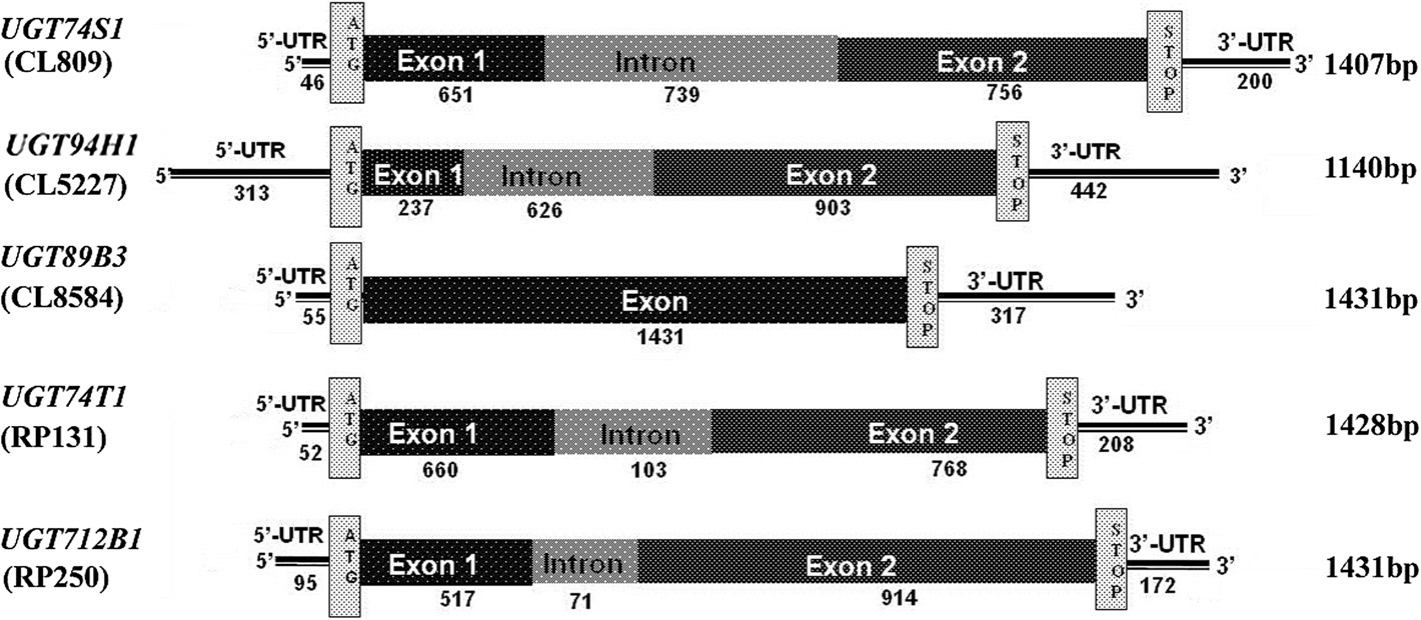
**Structural organization of the five flax UGT genes belonging to five sub-families.** Exons, introns and UTRs are illustrated with their respective length (bp) indicated below each region. The total length of the coding regions is shown on the right.

The structural organization of the 5 UGT genes was obtained using the flax WGS sequence assembly (Figure 2). The length of the UGT genes varied from 1597 bp to 2521 bp. Of the 5 flax genomic DNA regions corresponding to each of the full length UGT cDNAs, 4 had one intron, and one, *UGT89B3*, was intron free. All five were predicted to encode proteins of 379–476 amino acids. The intronic regions varied from 71 to 739 bp among the 5 UGTs whereas the exonic regions ranged between 237 to 1431 bp. The size of the amplified spliced cDNA for each of the 5 UGT genes (Additional file 2C) matched very closely with the exon size of the flax genomic DNA. The length of the 5′ un-translated region (5′ UTR) varied between 46 bp and 313 bp while the 3′ UTR ranged from 172 bp to 442 bp. Although showing the shortest spliced cDNA, *UGT94H1* appeared to be the largest UGT, with a size of 2521 bp (Figure 2).

### PSPG motif characterization

**Figure 3:**
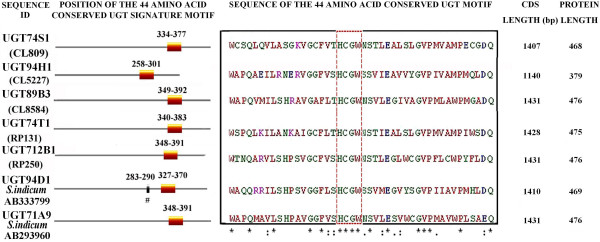
**Amino acid sequence alignment of the UGT PSPG conserved motif for five flax and two sesame UGTs.** The aldehyde dehydrogenases glutamic acid active site at position 283–290 is indicated with a # symbol *in S. indicum* UGT84D1.

Using the ExPASy PROSITE scan tool, the position of the PSPG conserved motif at the C-terminal of the open reading frame (ORF) was determined. The ORF of all five flax UGTs displayed the PSPG-box that is characteristic of UGTs’ family 1 (Figure 3). The conserved motif of 44 amino acids contains the tetra amino acid sequence HCGW, the most conserved signature among all the families. The 12 amino acids flanking the HCGW region of flax UGT94H1 showed 75% identity (9/12 flanking amino acids) with that of sesame lignan glycosylation *UGT94D1* gene (BAF99027.1), and an overall 66% identity over the 44 amino acids of the PSPG. Similarly, the PSPG of the flax UGT UGT89B3 shared an overall 64% identity with the sesame lignan glycosylation gene *UGT71A9* (BAF96582.1) and a 66% identity among the 12 amino acids flanking the HCGW region. The identity between 12 amino acids flanking the HCGW region of UGT74S1 and that of the sesame UGT71A10 (BAF96583.1) on one hand, and between UGT74S1 and UGT94D1 (BAF99027.1) on the other hand was 75 and 42%, and with an overall identity of 52 and 43%, respectively. Among the UGTs, higher variations were observed at the N-terminal region than at the C-terminal after a ClustalW multiple sequence alignment of the deduced amino acid sequences (Additional file 3).

### Tissue-specific *in silico* EST analysis of UGTs

A BLASTn search against the flax EST database that includes libraries from 13 different tissues revealed a higher level of expression in embryo and seed coat (Additional file 4). *UGT712B1* expression was exclusively detected in the globular and heart stage embryos (GE and HE) whereas *UGT94H1* was expressed in the torpedo (TE) and cotyledon stage embryos (CE), as well as in the torpedo stage seed coat (TC) (Additional file 4). *UGT74S1*, *UGT74T1* and *UGT89B3* were found exclusively in globular (GC) and torpedo stage seed coat (TC). *UGT74S1* and *UGT74T1* were the most abundant with 25 EST hits each in the TC EST library.

### Quantitative expression of UGTs and PLR in developing flax seed, leaf and stem tissues

**Figure 4:**
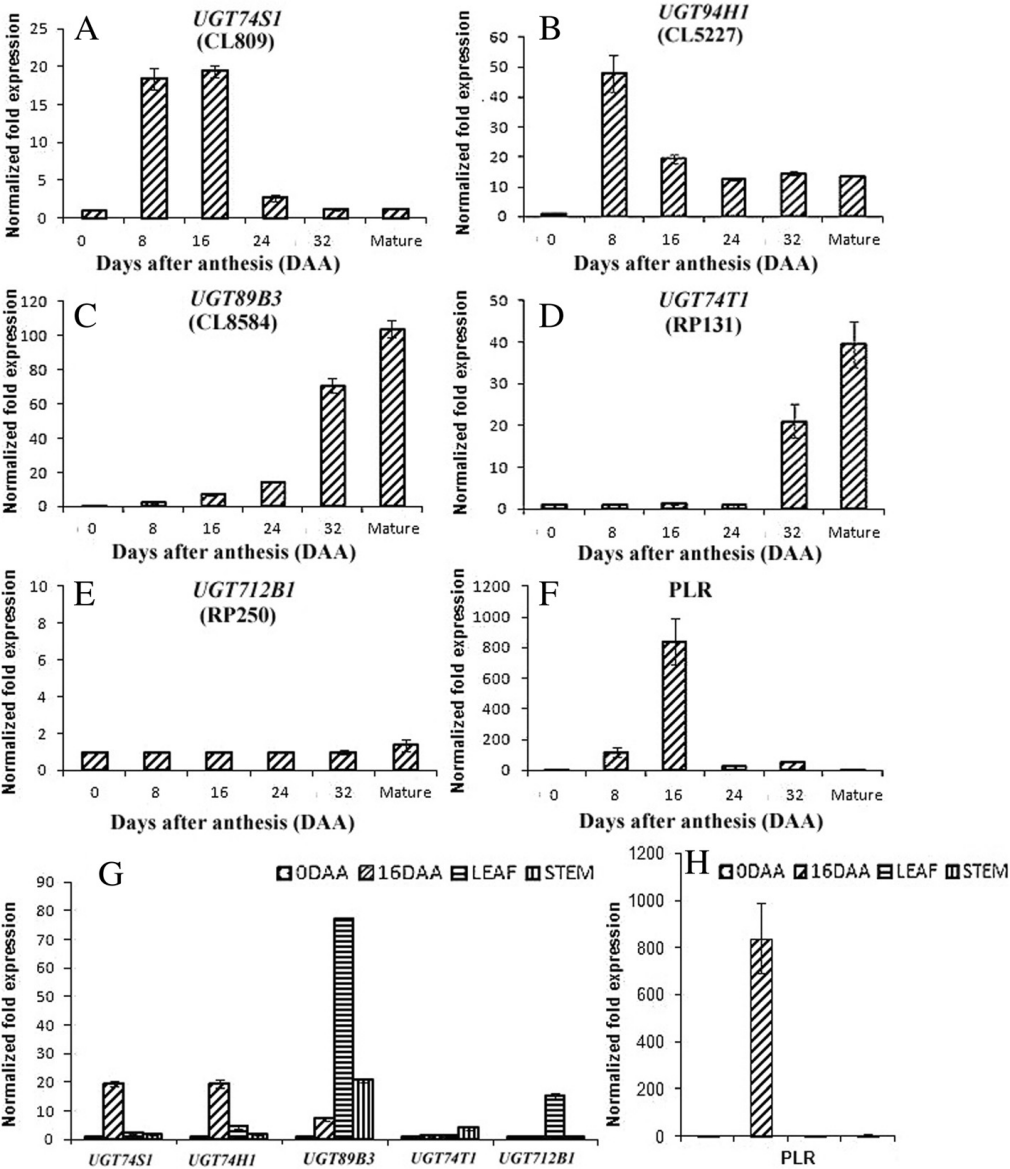
**Gene expression profile for the five UGT and one PLR genes in developing seed sampled at 0, 8, 16, 24, 32 DAA and at maturity as well as in leaves and stems of flax cultivar AC McDuff. A-F,** expression profile in developing flax seeds at six developmental stages; **A**, *UGT74S1*; **B**, *UGT94H1*; **C**, *UGT89B3*; **D**, *UGT74T1*; **E**, *UGT712B1*; **F**, *PLR*; **G**, expression of *UGT74S1*, *UGT94H1*, *UGT89B3*, *UGT74T1* and *UGT712B1* in flax seeds at 0 and 16 DAA, in leaves and in stems; **H**, expression of *PLR* at the same stages as G flax seed at two developmental stages (0 and 16 DAA) and in flax leaf and stem. The expression data were normalized relative to the reference gene at a linear scale averaged over three independent replicates and expressed as normalized fold change. Vertical bars represent standard deviation of the means.

Gene expression of the five UGTs and one PLR of flax cultivar AC McDuff differed for the different genes, amongst tissues and developmental stages (Figure 4A-H). In developing seeds, *UGT74S1* expression followed a bell curve pattern with peak expression at 16 days after anthesis (DAA) (Figure 4A). *UGT94H1* expression peaked at 8 DAA, declined at 16 DAA, and maintained a relatively stable expression afterwards until maturity (Figure 4B). *UGT89B3* showed an exponential increase of expression from 0 DAA to maturity (Figure 4C). *UGT74T1* was expressed at a low level between 0–24 DAA followed by a sharp increase at 32 DAA and at maturity (Figure 4D). *UGT712B1* was expressed at low and stable levels across all six seed developmental stages (Figure 4E). Low levels of expression were observed for *UGT74S1* and *UGT94H1* in the leaf and stem tissues. In contrast, *UGT89B3* was highly expressed in both vegetative tissues as compared to 16 DAA seeds. The expression of *UGT74T1* was higher in stems while that of *UGT712B1* was higher in leaves compared to other tissues (Figure 4G). The PLR expression pattern was similar to that of *UGT74S1* with peak expression at 16 DAA and no expression in leaf and stem tissue (Figure 4F and H).

#### SDG lignan profiling

**Figure 5:**
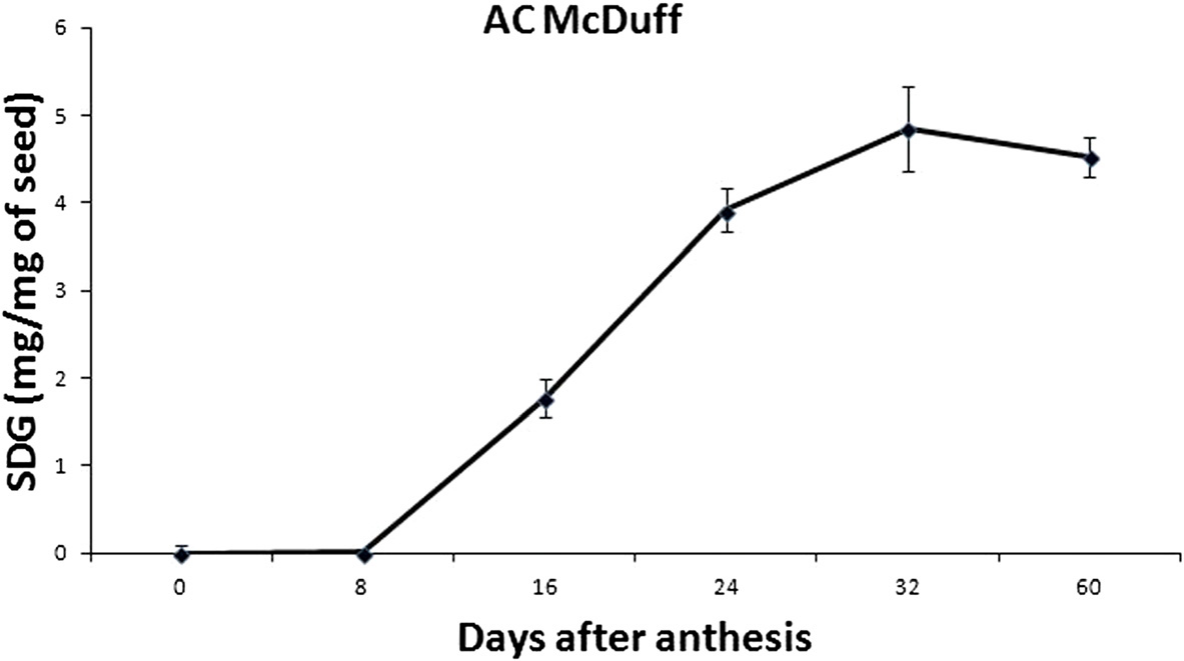
**Post-hydrolyzed SDG content during flax seed development.** The graph represents means from three replicates. Vertical bars are standard deviations of the means.

SDG lignan biosynthesis was assessed at six seed developmental stages of flax cultivar AC McDuff. The SDG lignan level was negligible between 0 and 8 DAA where a coniferin-like compound constituted the major metabolite observed at these stages (data not shown). The SDG lignan steadily increased starting at 8 DAA until 24 DAA when it started to plateau (Figure 5).

### Heterologous expression of flax UGTs and enzyme activities

**Figure 6:**
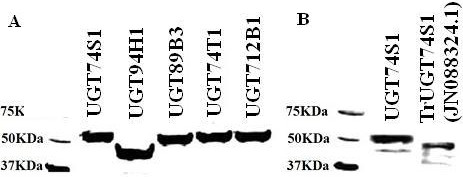
**Western blots of His Tag-purified proteins for (A) five UGTs and (B) UGT74S1 and a truncated form encoded by accession number JN088324.1 (TrUGT74S1) **[27]**using antiXpressTM antibody.** M, Western C precision plus protein marker mixed with conjugant (BioRad).

To ascertain a functional role for each of the five UGTs in SDG lignan biosynthesis, their full length cDNAs were expressed in yeast. All five proteins were highly expressed after eight hours of induction with 2% galactose and the molecular weight of the expressed proteins along with the Histidine-Tag were 56.4 kDa for UGT74S1, 46.2 kDa for UGT94H1, 55.9 kDa for UGT89B3, 56.4 kDa for UGT74T1, and 56.5 kDa for UGT712B1, in agreement with their predicted sequences (Figure 6A). Following the release of the flax draft genome, a flax UGT (GeneBank accession # JN088324.1) was reported [27]. This UGT clone is 100% identical to UGT74S1 at the amino acid and nucleotide levels but is predicted to be 150 nucleotides (50 amino acids) shorter at the 5′ end than UGT74S1 (*Lu*-UGTCL809) reported here (Additional file 5). For functional comparison purposes, a cDNA derived from UGT accession number JN088324.1 was also cloned and expressed in yeast. As expected, a smaller peptide of only 47 kDa was observed compared to 56.4 kDa *for* UGT74S1 (Figure 6B). The gene corresponding to JN088324.1 is hereafter referred to as truncated *UGT74S1* (*TrUGT74S1*).

### Enzyme assays and reactions conditions

**Figure 7:**
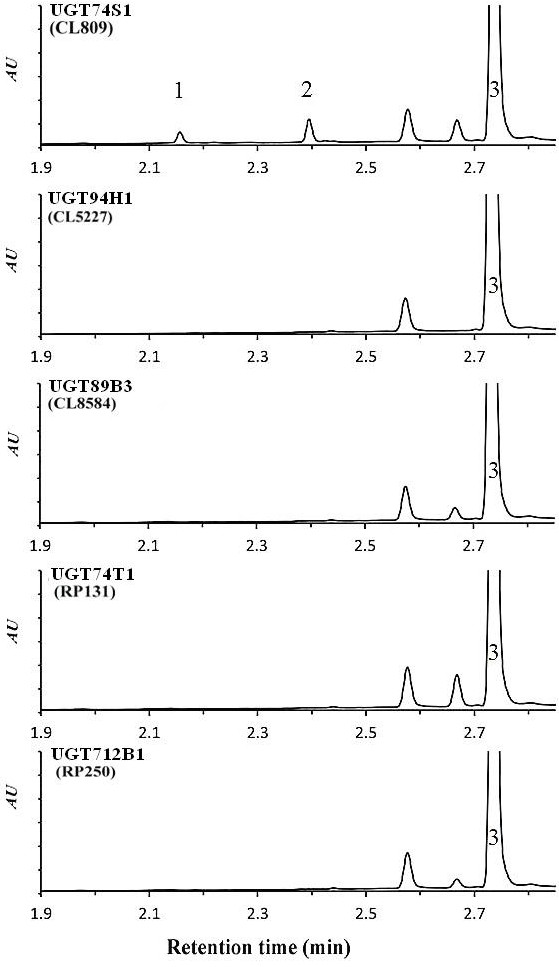
**UPLC chromatograms showing the reaction products of 50 μg of crude proteins for five UGTs using SECO.** Each chromatogram corresponds to the reaction profile for the enzyme indicated. Peaks 1 and 2 were observed only in chromatograms of UGT74S1 along with the unreacted SECO peak 3 present in all chromatograms.

To identify the flax UGTs potentially involved in SECO glycosylation, 50 μg of crude recombinant protein for each of the 5 UGTs expressed in yeast was assayed with different aglycones including secoisloariciresinol, sillibinin, quercetin, kaempferol, coumaric acid, caffeic acid, sinnapic acid, cinnamic acid and ferulic acid (data not shown). Only UGT74S1 exhibited an activity by producing two new peaks using only SECO as a substrate (Figure 7).

**Figure 8:**
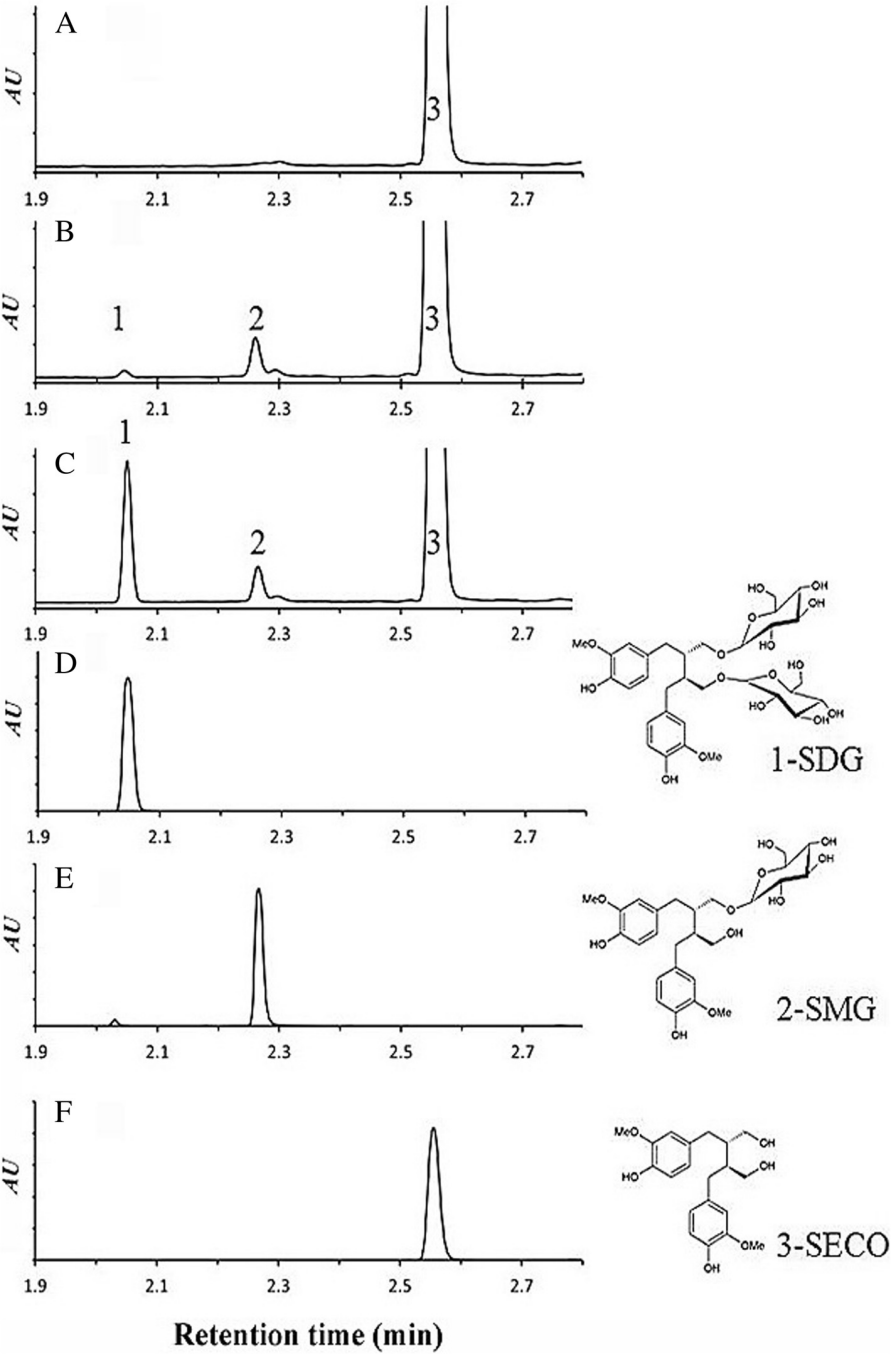
**UPLC chromatograms identifying the reaction products of UGT74S1 with SECO as SDG (peak 1) and SMG (peak 2). A**, negative control including reaction buffer, SECO, UDP-glucose, and no enzyme; **B**, enzyme reaction including reaction buffer, SECO, UDP-glucose, and 50 μg of crude UGT74S1 enzyme. Peaks 1, 2, and 3 refer to the SDG, SMG and SECO peaks, respectively; **C**, enzyme reaction spiked with SDG standard prior to UPLC analysis **D**, SDG standard; **E**, SMG standard; **F**, SECO standard. The structures for SDG, SMG, and SECO are shown on the right.

To confirm the identity of the observed peaks, the enzyme reaction was spiked with SDG and resolved alongside various controls and standards (Figure 8). A negative control without enzymes (Figure 8A), positive controls with standard SDG (Figure 8D), positive controls with standard SMG (Figure 8E) and standard SECO (Figure 8F) were included. The detected SMG peak 2 was higher than the detected SDG (peak 1) (Figure 8B). The identity of the small peak 1 was confirmed by spiking a known amount of standard SDG to the reaction products prior to UPLC analysis; the resulting peak increased in size and eluted with an identical retention time as the standard SDG (Figure 8C and D). Thus, glucosylation of SECO into SMG primarily, and SDG to a smaller extent, occurred in the presence of UGT74S1 (Figure 8).

**Figure 9:**
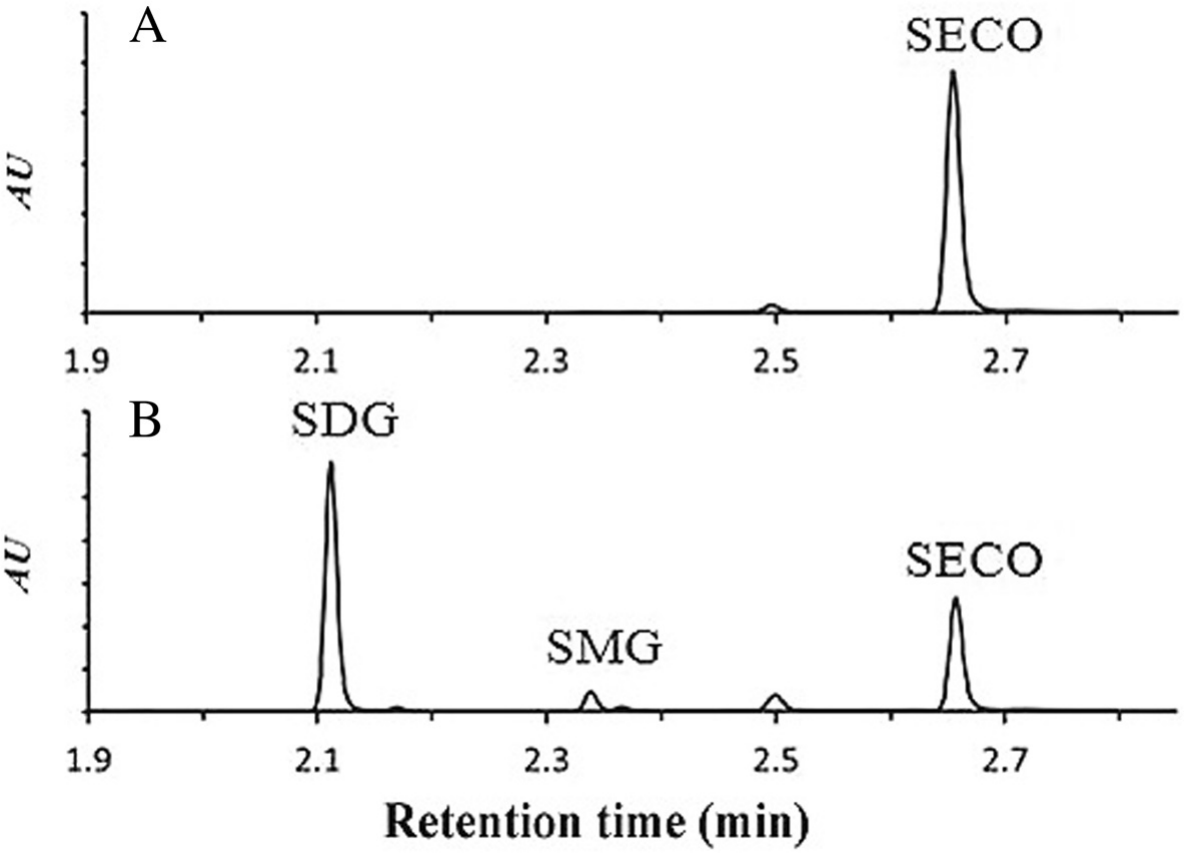
**UPLC Chromatograms shows a higher production of SDG compared to SMG from affinity-purified UGT74S1 protein using SECO as a substrate. A**, Negative control consisting of reaction buffer, SECO, UDP-glucose and no enzyme; **B**, Enzymatic reaction products of SECO and UDP-glucose using 50 μg of His tag-purified UGT74S1 enzyme.

To ascertain these observations, the five enzymes were further purified using 6X His-tagged Nickel chelating purification system and 50 μg of the purified proteins were reacted with SECO. Similar to the crude protein, only the purified UGT74S1 showed the same two new peaks when SECO was used as a substrate (Figure 9A and B). Contrary to the reaction with the crude protein, the purified protein produced a higher SDG level compared to SMG (Figure 9B). Thus, enzyme purification enhanced SECO glycosylation into SDG by UGT74S1.

**Figure 10:**
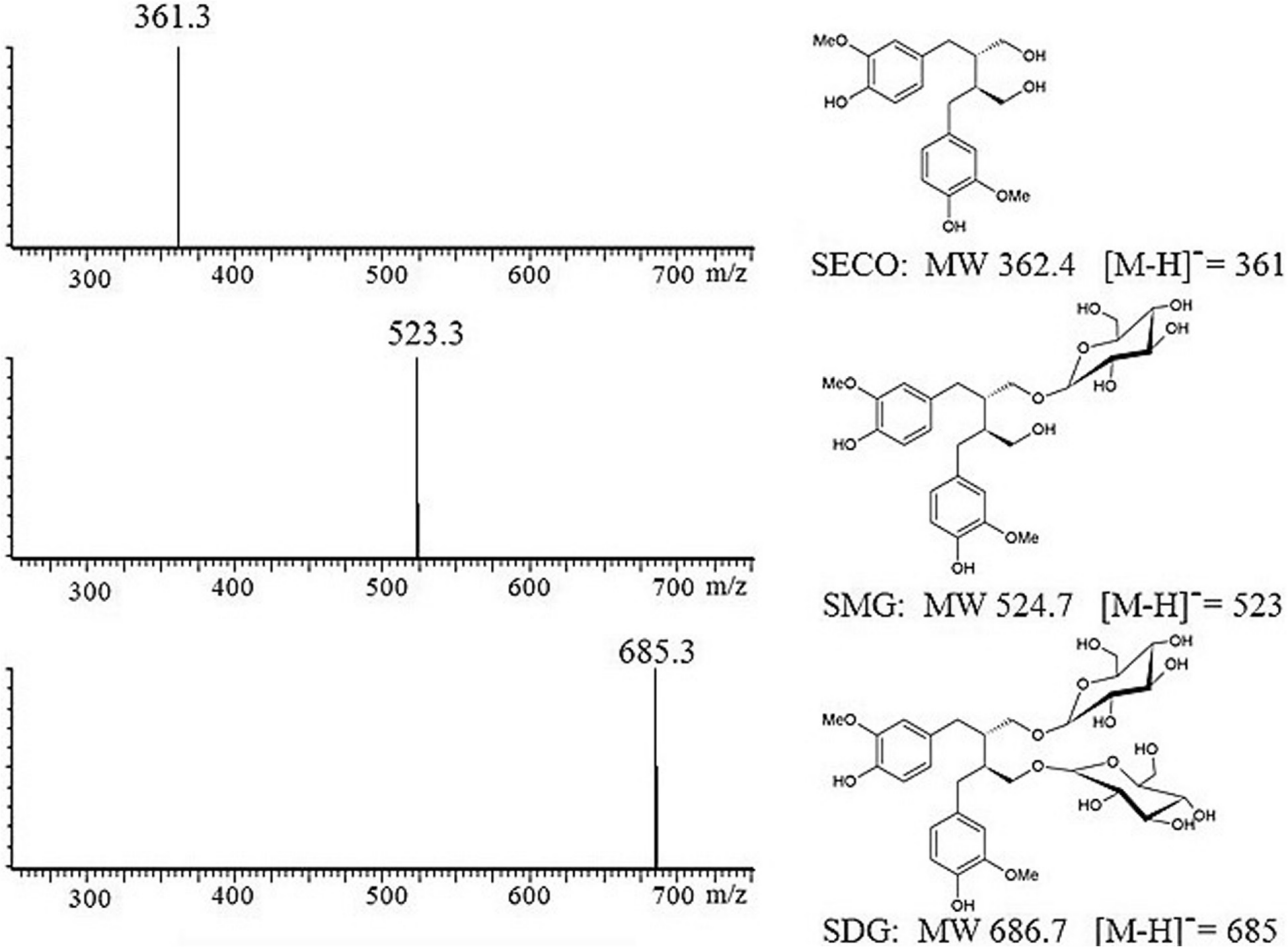
**LC-ESI-MS spectra of UGT74S1 enzyme reaction products with SECO.** The observed molecular weight for each metabolite (SECO, SMG and SDG) is shown next to its corresponding spectra. The expected molecular weights and [M-H]^+^ pseudomolecule ions are also shown under their respective structure.

Liquid Chromatography–Electrospray Ionization–Mass spectrometry (LC-ESI-MS) analysis allowed a better characterization of the *de novo* synthesized SMG and SDG. The two new products exhibited a molecular ion at mass-to-charge ratio (m/z) of 523 and 681 [M–H] ^-^ for SMG and SDG, respectively, consistent with their known MW (Figure 10). ^1^H, ^13^C correlation spectroscopy nuclear magnetic resonance (^1^H, ^13^C COSY) NMR experiments of the LC purified peaks 1, 2 and 3 confirmed their identities (data not shown), closely matching previous reports for these compounds [33].

### UGT74S1 biochemical parameters

**Figure 11:**
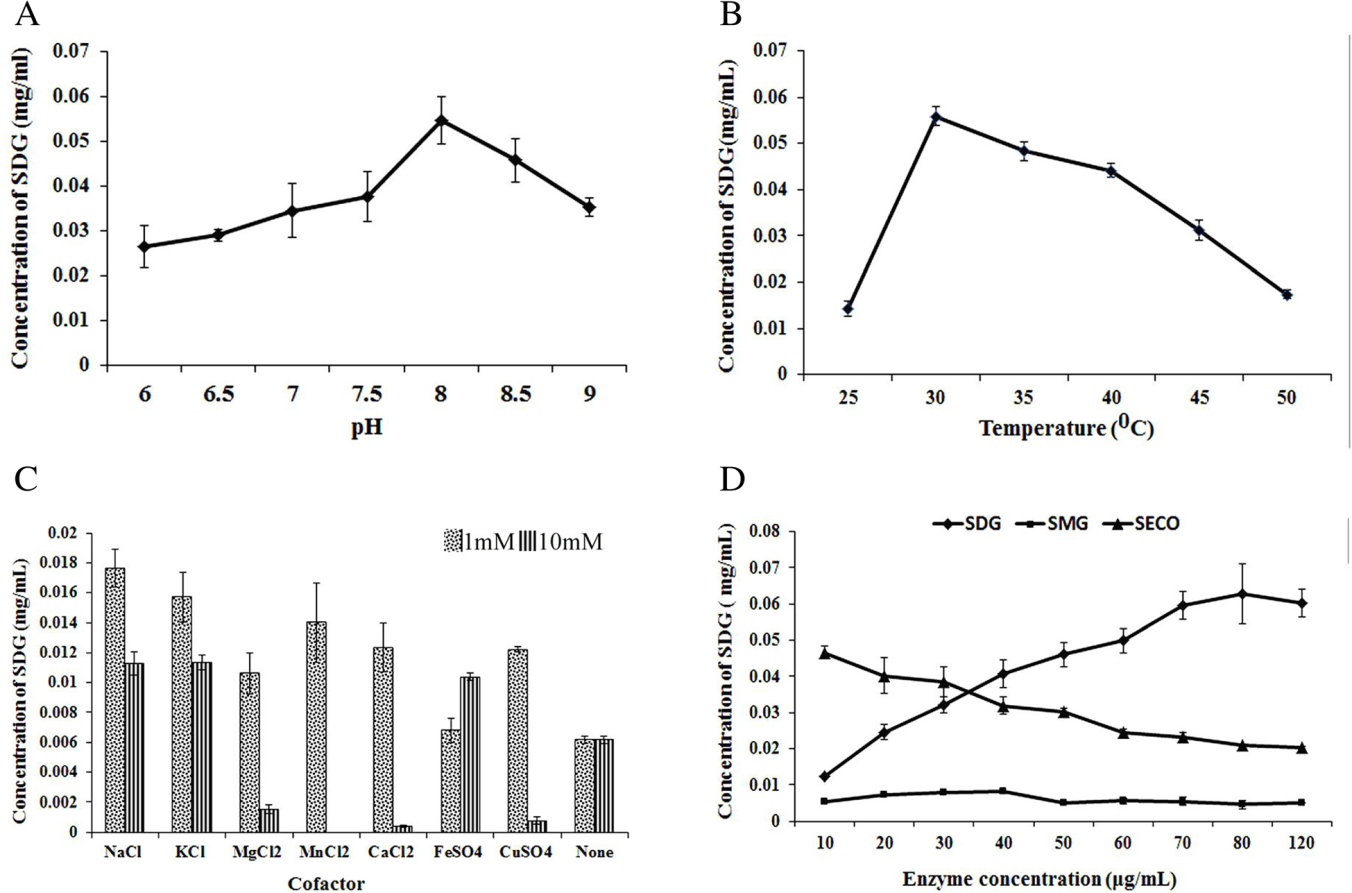
**Optimization of UGT74S1 reaction conditions. A**, Effect of pH; **B**, Effect of temperature; **C**, Effect of two concentrations of seven different metal cofactors; **D**, Effect of enzyme concentration.

Different pH ranges, temperatures, cofactors and enzyme concentrations were assayed to optimize the UGT74S1 reaction with SECO. The optimal pH was determined to be 8.0, with a low activity below pH 7.5 and at 9.0 (Figure 11A). Optimal temperature for UGT74S1 activity was at 30°C (Figure 11B). All the cofactors evaluated in this study activated the UGT74S1 enzyme at 1 mM, except for FeSO_4_ which activated at 10 mM (Figure 11C). A concentration of 10 mM MgCl_2,_ MnCl_2_, CaCl_2,_ or CuSO_4_ inhibited UGT74S1 activity. Of the cofactors tested, NaCl was the most effective catalyst (Figure 11C). Increased concentration of UGT74S1 from 10–120 μg increased activity up to 80 μg, after which a saturation effect was observed (Figure 11D). These optimal biochemical parameters (pH 8.0, 30°C, 1 mM NaCl, and 80 μg proteins) were subsequently used in the rest of the study.

Because UGT94H1, UGT89B3, UGT74T1 and UGT712B1 did not glycosylate SECO into SMG, further tests were conducted to determine if they were involved in the glucosylation of SMG to form SDG. Since SMG is not commercially available, SDG was hydrolyzed to SMG [33]. Using this SMG as a substrate, the five UGTs were assayed. But again, only UGT74S1 showed a peak corresponding to SDG retention time (data not shown). Therefore, UGT89B3, UGT74T1, UGT712B1, and UGT94H1 appeared not to be involved in SDG lignan glycosylation and their biochemical function remains to be elucidated. Thus, UGT74S1 was the only flax UGT cloned and identified in this study that used SECO as a substrate, first producing SMG and then SDG in a sequential manner. Its truncated version TrUGT74S1 was also assayed using the optimal conditions set for *UGT74S1* and was also unable to glucosylate SECO (Additional file 6).

### UGT74S1 kinetic parameters

By reacting UGT74S1 with SECO at pH 8.0 and 30°C, the catalytic efficiency (kcat) for SDG production was determined to be 0.89 sec^−1^. The estimated apparent Km values toward SECO and UDP-glucose for SDG production were determined to be 79 and 1188 μM, respectively.

## Discussion

UGTs are a large and complex family of enzymes that catalyze glycosidic bond formation. To get a better understanding of UGTs that may play a role in the glycosylation process of flax SDG lignan, we undertook the cloning and characterization of flax UGTs. We identified and characterized five flax full length UGTs, namely *UGT74S1*, *UGT94H1*, *UGT89B3*, *UGT74T1*, and *UGT712B1*. We found that *UGT74S1* and *UGT94H1* were highly expressed in developing seed and their expression was coordinated with that of *PLR*, the first-step lignan biosynthetic gene [29], and well correlated with the SDG lignan biosynthesis patterns in seed. By expressing each of the five UGTs and reacting the purified proteins with SECO and UDP-glucose, only UGT74S1 produced both SMG and SDG metabolites. To our knowledge, this is the first demonstration linking any flax UGT gene to SDG lignan biosynthesis.

The International Union of Biochemistry and Molecular Biology and IUPAC-IUBMB joint committee responsible for UDP-glycosyltransferase [32] classified the five UGTs into four families and five sub-families, representing five distinct genes. In the course of this study, Barvkar et al. [27] probed the recently released flax genome ([26]; Deyholos, http://www.linum.ca) and reported 137 flax UGTs including homologs to our reported *UGT74S1* (*CL809*), *UGT94H1* (*CL5227*), *UGT89B3* (*CL8584*) and *UGT712B1* (*RP250*). These were not, however, characterized with regards to their functionality towards aglycones. Moreover, TrUGT74S1 (JN088324.1; [27]) was 50 amino acids shorter than UGT74S1 described herein (Additional file 5). We provided convincing evidence that TrUGT74S1 is unable to glucosylate SECO into SDG, and is thereby not functional (Additional file 6). The 50 amino acids missing in TrUGT74S1 seem to be essential for glucosyltransferase activity.

The UGTs described in this study differed in their structural organization, primary sequence, and in their PSPG motifs. Coding sequence variation among plant UGT family 1 members is generally high, varying from less than 35% to more than 95% overall identity [34], with the C-terminal regions that contain the PSPG box being more conserved [24]. Although well conserved, diversity within the PSPG motif of the five flax UGT genes was revealed. At the structural level, one of the UGTs had no introns while the remaining four had one intron each, which varied in size from 71 to 739 bp. In *Arabidopsis*, more than half of the UGTs have no introns [24] and those with introns were much smaller (~100 bp), a difference somewhat proportional to the genome size differences of ~370 Mb for flax and 135 Mb for *Arabidopsis*. Differences were also observed in the spliced coding sequence (CDS) sizes (379 to 476 amino acids), further emphasizing the diversity within the UGT family and in agreement with its recent origin hypothesis [22,23].

Although UGT family 1 is a very diverse gene superfamily, its members are usually classified based on their sequence identity [35] and the presence of the conserved PSPG motif [34] that includes key conserved residues for substrate recognition and catalysis [6]. The UGTs described herein all possessed the conserved 44 amino acid PSPG motif and the two peptide sequences, W*AP*QV and HCGW*N*S, present in 95% of all β-group GTs analysed to date [34]. Amino acid variations were nonetheless observed (italized positions) in these two short peptide motifs as well as in the C-terminal of the PSPG-box [23]. Sugar donor specificity has been attributed to the PSPG box [17]. For example, substitution of tryptophan (W) at position 355 (position 22 of PSPG) for arginine (R) sufficed to modify the sugar donor specificity from UPD-glucose to UDP galacturonic acid in *Lamiale*[36]. The domain involved in the recognition and binding of the diverse aglycone substrates is purported to be located towards the N-terminal end, whereas the C-terminal region encodes a domain involved in binding the nucleotide sugar substrate [37].

**Figure 12:**
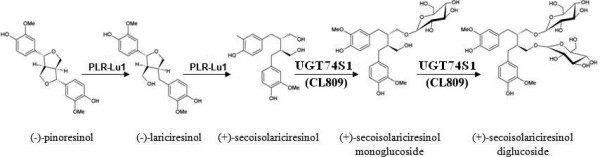
**Proposed model for secoisolariciresinol diglucoside (SDG) lignan biosynthesis in flax plants.** Secoisolariciresinol (SECO) undergoes sequential glucosylations by UGT74S1 that catalyzes both the first glucosylation of SECO to form secoisolariciresinol monoglucoside (SMG) and the second glucosylation of SMG to SDG.

Transcriptome analyses revealed that the flax UGTs reported here were expressed predominantly in embryo and seed specific libraries [38]. These results were validated and quantified by qPCR. The expression of *PLR*, *UGT74S1*, and *UGT94H1* appeared to be coordinated and correlated with SDG lignan accumulation in the seed. Despite the similar expression pattern of *UGT74S1* and *UGT94H1* and their correlations with SDG lignan accumulation in developing seed, only UGT74S1 was demonstrated to metabolize SECO, first into SMG and then into SDG lignan. Because free SMG has not yet been reported *in planta*, the occurrence of an enzyme that glucosylates only SECO or SMG cannot be ruled out in flax but would not be essential considering that UGT74S1 is capable of catalyzing the last two steps. Hence, we propose the following model for the sequential glucosylation of SECO by UGT74S1 to form SDG via a SMG intermediate (Figure 12).

The optimal enzyme conditions (pH, temperature, cofactors) for UGT74S1 were established and fall within the range of the majority of UDP-glycosyltransferases [39]. UGT74S1 was found to be sensitive to increased ionic strength of metal ions as reported for other UDP-glycosyltransferases [39,40]. The UGT74S1 apparent Km for UDP–glucose was higher than that for SECO, and fall in the Km ranges previously reported [41-43]. The catalytic efficiency (k_cat_) of UGT74S1 for SECO was close to that of UGT71A9 and UGT94D1 reported by Noguchi et al. [6]. None of the flavonoid or phenolic acid aglycone substrates tested in this study served as good substrates for UGT74S1.

## Conclusions

Taken together, we have cloned five UGTs from flax seeds and demonstrated through a comprehensive multi-approach analysis that *UGT74S1* was a functional enzyme capable of converting SECO into SDG. Our results suggest that *UGT74S1* is involved in secoisolariciresinol glucosylation *in planta* to form flax SDG lignan. The findings shed more clarity in flax lignan biochemistry and provide the necessary background to conduct site directed mutagenesis studies.

## Methods

### Plant materials

Flax plants (*Linum usitatissimum* L. cv AC McDuff) were grown at AAFC Harrington farm (Harrington, PEI, Canada) in the 2008 to 2011 growing seasons. Plants were grown in four replications each year. At anthesis, referred to as 0 days after anthesis (0 DAA), individual flowers were tagged. Developing bolls were harvested at 0, 8, 16, 24, 32 DAA and at maturity and immediately frozen in liquid nitrogen as previously described in Arabidopsis [44], soybean [45] and flax [46,47]. The 0 DAA samples consisted of ovaries free of other flower tissues, whereas the other boll samples (8–32 DAA and maturity) contained seeds at different developmental stages (Additional file 7). At the flowering stage, young leaf and stem tissues were similarly collected. Developing bolls, leaves and stems were stored at −80°C until use.

### RNA isolation

Before RNA isolation, ovules (0 DAA) and developing seeds were first extracted from the bolls. Total RNA was isolated using Trizol (Invitrogen, Carlsbad, ON, Canada) as previously described [46]. RNA samples were further purified using the Invitrogen PureLink™ RNA Mini kit (Invitrogen, Mississauga, ON, Canada) as per manufacturer’s instructions, quantified by spectrophotometry, and the quality was verified by agarose gel eletrophoresis and the Experion RNA analyzer (BioRad, Missisauga, ON, Canada).

### Library mining and UGT cloning

The flax NAPGEN EST database (Plant Biotechnology Institute, NRC, Saskatoon) was mined using the keywords UGT, glucosyltranferase and glycosyltranferase. A total of 893 UGT hits were found amongst 178,656 ESTs. For primer design, we retained members of UGT subclasses 71 (7 hits), 88 (3 hits) and miscellaneous (7 hits). A set of 19 flax-specific and one degenerated primer pairs were designed (Additional file 8).

Total RNA (2 μg) from all developmental stages was used as template to create the cDNA using the first strand cDNA synthesis kit (Invitrogen, Mississauga, ON, Canada) following manufacturer’s instructions. After treatment with 2 U RNAse H (Invitrogen), the cDNA samples were diluted 10-fold and 1 μL was used as template. Each of the 20 primer pairs (Additional file 8) was used in PCR reactions consisting of an initial denaturation 94°C for 2 min followed by 35 cycles of 94°C for 30 s, 60-63°C for 30 s, and 72°C for 60 s prior to a final extension at 72°C for 10 min. Aliquots of 10 μL of the PCR products were resolved on 1% agarose gels stained with ethidium bromide. The amplified fragments were purified using the QIAquick gel extraction kit (Qiagen) for direct sequencing and for TOPO cloning (Invitrogen) in *E. coli* prior to sequencing.

The identities of the obtained partial sequences were confirmed by BLASTx against the NCBI non-redundant protein sequence (nr) database using a cut off value of 1e^−30^. The relationship between the partial sequences was inferred by a phylogenetic consensus tree constructed using UPGMA method with 1000 bootstrap replicates as implemented in MEGA4 [48].

To clone the full length UGTs, 5′ and 3′ gene specific primers (GSP) and nested gene specific RACE PCR primers were designed from representative sequences of each group observed in the consensus tree (Additional file 1) and were used in 5′ and 3′ cDNA end amplification reactions. Briefly, using the Gene Racer kit (Invitrogen, Mississauga, ON, Canada), the purified total RNA was dephosphorylated using a calf intestinal phosphatase, and decapped with a tobacco acid pyrophosphatase. The RNA oligos were ligated to the decapped mRNA by T4 RNA ligase (Invitrogen, CA, USA) before reverse transcription of mRNA using oligo-dT primers. The 5′ and 3′ RACE PCR reactions were carried out using eight pairs of GSP and nested primers (Additional file 9) following the kit’s specifications. The expected 5′ and 3′ RACE PCR products of the putative UGTs CL809, CL5227, CL8584, RP131, RP250 were gel-purified, cloned in TOPO 4.0 vector (Invitrogen, Mississauga, ON, Canada) and sequenced using M13 forward and reverse primers. New primer sets containing restriction sites compatible with the multiple cloning site of pYES2/NT C plasmid vector (Invitrogen, Mississauga, ON, Canada) were designed from the 5′ and 3′ ends (Additional file 10) for the amplification of the full length cDNAs (Additional file 2). The amplified full length cDNAs were gel-purified, restriction digested and similarly cloned into pYES2/NT C. The cDNA corresponding to one of the UGT clones reported by Bavkar et al. [27] (accession JN088324.1) was also cloned as described above. The plasmids carrying the full length cDNA clones were sequenced using T7 promoter primer x(5′-TAATACGACTCACTATAGGG-3′) and CYC1 reverse primer (5′-GCGTGAATGTAAGCGTGAC-3′).

### UGT structural gene organization

To characterize the structural organization of the flax genomic DNA corresponding to each of the five UGTs, a BLASTn search within the flax sequence assembly (http://www.linum.ca) was performed to identify the 5′ and 3′ untranslated regions (UTRs), and the intron and exon structure of the coding regions. The PROSITE scan tool of the ExPASy web interface (http://www.expasy.org) was used to determine the position of the conserved motifs characteristic of plant UGTs such as the PSPG box.

### *In silico* analysis of UGTs

To characterize the relative abundance of the cloned UGTs, an *in silico* EST analysis was performed. The five full length UGT sequences were compared to 13 flax tissue-specific EST libraries (globular embryo, heart embryo, torpedo embryo, cotyledon embryo, mature embryo, pooled endosperm, globular stage seed coat, torpedo stage seed coat, etiolated seedling, leaves, stem, stem peel and mature flower) previously described [38] and the number of EST hits corresponding to each query UGT in each library was recorded and plotted.

### UGT real time gene expression analysis

To assess the gene transcript expression levels of the putative cloned UGTs in developing flax seed, leaf and stem tissue, real-time PCR primers were designed from the five flax UGTs, one PLR and one ribosomal (EU307117) RNA sequence (Additional file 11). The rRNA primers were used for data normalization. Total RNA was extracted from three separate biological replicates for each seed developmental stage (0, 8, 16, 24, 32 DAA, and mature seed). First strand cDNA was obtained as described earlier. The cDNA samples were quantified by spectrophotometry or Qubit (Invitrogen) and diluted to 100 ng/μL. Real-time PCR reactions were performed using the SYBR Green PCR Master Mix (BioRad Laboratories, Canada) on a CFX96 Real Time system (BioRad). For each sample, three biological and three technical replicates, for a total of 9 data points, were obtained. The 25 μL Real Time amplification reactions consisted of 1x SYBR Green Master Mix, 300 nM of each primer, 100 ng of first strand synthesis cDNA obtained from ovaries (0 DAA), developing seeds (8, 16, 24, 32 DAA), mature seeds, leaves, stems and water controls. Real-time PCR reactions were performed as follows: denaturation at 95°C for 10 min followed by 40 cycles of 95°C for 30 s, 60°C for 30 s. Following the final amplification cycle, a melting dissociation curve was generated to ensure specificity of the primers and to confirm the uniqueness of the amplification product. The output data was determined following the 2^-∆∆CT^ method described by Livak and Schmittgen [49] and it is reported as fold changes of relative expression.

### SDG lignan profiling in developing flax seeds

To assess the SDG lignan biosynthesis in developing flax seeds, 250 mg of ovary or seed at six developmental stages was used as starting material following modifications to a protocol described by Popova et al. [50]. Developing flax seed tissue was ground to a fine powder in liquid nitrogen using mortar and pestle. The powder (200 mg) was transferred into a glass centrifuge vial and defatted with 2 mL hexane (1:10 w/v) on a Wrist Action Shaker (Burrell Scientific, PA, USA) for 2 h at room temperature. After centrifugation at 1500 rpm for 15 min, the supernatant was discarded. The pellet was rinsed with 2 mL hexane, centrifuged and air-dried for 15 min. The defatted material was extracted with 2 mL of 70% (v/v) methanol/water at 55°C for 2 h using rotation in an oven, with intermittent manual shaking 2–3 times. A final vigorous shaking was performed for 15 min on a Wrist action shaker (Burrell Scientific, Pittsburgh, PA, USA) at room temperature. The samples were centrifuged at 1500 rpm for 15 min and the supernatant (S1) was collected in new capped vial. The residue was rinsed again with 0.5 mL 70% methanol, centrifuged and the supernatant (S2) was collected and pooled with S1. The total supernatant volume was recorded before hydrolysis. The combined samples (S1 + S2) were hydrolysed for 1 h at 60°C with 0.5 N NaOH at a ratio of 3:5 (v/v). After hydrolysis, the samples were immediately neutralized using 0.5 N HCl at a ratio of 0.4 mL for every 0.5 mL extract. The hydrolysate was cooled and purified via solid phase extraction using 10 mL Waters HLB columns (Waters, Mississauga, ON, Canada). The eluted lignan fractions were collected in glass vials and dried using a rotary evaporator (Heidolph instrument Gamborg, Germany). The dried material was dissolved in methanol:water (50:50), filtered and injected for UPLC-MS analysis using a commercially available SDG standard (Chromadex, Irvine, CA, USA) as reference.

An Acquity H-Class, quaternary pump UPLC system (Waters, Mississauga, ON, Canada) equipped with in-line degassing, diode array detector (DAD), robotic autosampler, sample and column temperature controls and tandem quad mass spectrometer (TQD) was used for lignan profiling analysis. A ternary solvent system for UPLC-MS analysis consisting of water, acetonitrile and 10% formic acid in water was used for UPLC-MS analysis. UV–vis spectra were recorded from 210–600 nm, and the MS was run in ESI mode, 3000 V capillary voltage, in scanning mode from 100–2000 a.m.u., with a fragmentation setting of 150 V, 13.0 L/min carrier gas (N_2_) flow at 350°C and 60 psi to ensure identity of the profiled metabolites. The post-hydrolysis SDG lignan peak was identified and quantitated through comparison (UV–VIS absorption, retention time) to a commercial standard. Other phenolic compounds, including hydroxycinnamic acids liberated by the base hydrolysis were present but were not quantified. A standard curve for SDG was created, relating integrated peak area (mAU*s) (Y) versus concentration of SDG (mg/mL) (X). In brief, 1 mg of authentic standard was dissolved in 50% methanol and a serial dilution was created in triplicate, halving the concentration each time. The resulting standard curve was linear from 0.5 mg/mL to 0.00781 mg/mL (R^2^ = 0.9901) and was used to determine SDG content in relation to developmental stage (DAA). For each of the six developmental stages, three extractions and HPLC analyses were performed from three biological replicates and the values were presented as the mean of the three data points.

### Heterologous expression of flax UGTs in yeast

The pYES2/NT C plasmid constructs harbouring the cDNA of the five UGTs described in this study were used to transform yeast INVSc1 strains using *S.c.* EasyComp transformation™ kit (Invitrogen, CA, USA). The flax UGT cDNA of Genebank accession JN088324.1 [27] was similarly transformed for functional comparative analyses. Single transformant INVSc1 yeast colonies were inoculated into 15 mL of *Saccharomyces cerevisiae* minimal media without uracil (SC-U, prepared as recommended by Invitrogen) supplemented with 2% raffinose and grown for 3 days under shaking at 30°C until the OD_600_ reached 2.0. The culture was diluted in 50 mL of induction medium (SC-U supplemented with 1% raffinose and 2% galactose) to achieve an initial OD_600_ of 0.4. The culture was further incubated under shaking at 30°C for 24 hours, with 5 mL sub-sample collection at 0, 4, 8, 12 and 24 h to monitor the protein expression. The OD_600_ for each time point was recorded. The induced yeast cells were harvested by centrifugation at 1,500 *g* for 5 min at 4°C. The cells were washed using 500 μL cold sterile distilled water and centrifuged. The pellets were washed again at 4°C in 500 μL of lysis buffer (50 mM sodium phosphate, pH 7.4 supplemented with 5% glycerol and 1 mM PMSF). After centrifugation, the cells were mechanically disrupted by vortexing for 30 seconds in the presence of an equal volume of 425–600 μm acid-washed glass beads (Sigma Aldrich, Canada). After vortexing, the sample was incubated on ice for 30 seconds. The vortexing and incubation cycle was repeated 4 times to ensure complete cell lysis. The lysates were centrifuged at 18,620 *g* for 10 min at 4°C and the supernatant was collected. The optimum induction time for all the UGTs was monitored by western blot using equal amount of proteins and antibodies raised against the anti-ExpressTM epitope present between the 6x Histidine tag and the multiple cloning site of the construct. The polyhistidine containing recombinant proteins was purified using the ProBond™ (Invitrogen, CA, USA) purification system following manufacturer’s instruction. The purified enzymes were concentrated using 0.5 mL Ultracel^R^-10 k Amicon membrane column (Millipore, Ireland). Protein concentrations were determined using the Bradford protein assay kit (BioRad Laboratories, Canada).

### Enzyme assays

The crude and purified recombinant protein extracts obtained from the yeast cultures harboring the five different UGT cDNAs reported in this study and the one derived from JN088324.1 [27] were reacted with different aglycone substrates including SECO (Chromadex, Irvine, CA, USA), sillibinin, quercetin, kaempferol and the phenolic acids coumaric acid, caffeic acid, sinnapic acid, cinnamic acid, and ferulic acid (Sigma Aldrich, Canada). The 100 μL reaction mixture consisted of a reaction buffer (50 mM sodium phosphate, 1 mM PMSF, 5% glycerol, pH 7.4), 280 μM aglycone substrate (acceptor for glycosylation), and 1.64 mM UDP-glucose (sugar donor) (Sigma Aldrich, Canada). The reaction mixtures were pre-incubated at 30°C for 10 min and the reactions were initiated with the addition of 50 μg of enzyme. After incubation at 30°C for 30 min, the reactions were stopped with 100 μL of 0.5% trifluoroacetic acid in acetonitrile. The reaction mixtures were purified using 0.2 μm filters (Pall Life Sciences, Mississauga, ON, Canada) to remove any particulates that might form during the reaction. The separation and identification of the reactants and products derived from the enzyme assays were carried out using a Waters H-Class Acquity UPLC system (Waters, Missisauga, ON) equipped with a TQD tandem mass spectrometer. The formation of glycosylated products was monitored by examining the masses and the principle fragments of eluted peaks via ESI–mass spectrometry. Two parallel MS2 scans were performed ranging from 120–800 a.m.u., using 15 and 45 V cone voltages. Selected ion recording (SIR) spectra were also collected to enhance the sensitivity of detection of SECO, SMG and SDG. The capillary voltage was 3 kV, the extractor set to 3 V, and RF lens at 0.1 V. Chromatographic conditions consisted of a binary gradient system composed of 3% formic acid in water (A) and acetonitrile (B), varied according to the following gradient: t0, A = 68%; t1 = 4.4 min, A = 0%; t2 = 6 min, A = 0% isocratic; t3 = 7 min, A = 68%; t4 = 8 min, A = 68% isocratic. Peaks detected at 280 nm, indicative of phenolic compounds, were validated using authentic standards (SECO and SDG) purchased from Chromadex (Chromadex, Irvine, CA, USA). A standard curve for SDG was created as detailed above. Standard purified SMG was prepared as described by [33].

### Kinetic and biochemical characterization of UGT74S1

Ranges of pH from 6.0 to 9.0, temperature from 25°C to 50°C, enzyme concentration from 10 to 120 μg and two concentrations (1 and 10 mM) of seven metal cofactors (NaCl, KCl, MgCl_2_, MnCl_2_, CaCl_2_, FeSO_4_ and CuSO_4_) were tested in 100 μL reaction mixture for determining the optimal pH, temperature, enzyme concentration and metal cofactor effect on the enzyme activity. To determine the initial velocity of the recombinant *UGT74S1* enzyme, a time course (5, 10, 15, 30, 45, 60 min) study using the optimum enzyme concentration and fixed excess substrate (280 μM SECO; 1.67 mM UDP-glucose) concentration was conducted at 30°C, pH 8. The linearity was maintained in assays up to 30 min at 30°C. The initial velocity of the reaction was measured at 10 min, where no more than 10% of SECO was converted to SDG at this time point. Then, the assays were carried out using various substrate concentrations (70–1400 μM SECO with UDP-glucose fixed at 1.67 mM; 0.82–6.56 mM UDP-glucose with SECO fixed at 280 μM), under optimum conditions, for 30 min, for the determination of kinetic parameters. The apparent *V*max and *K*m value for the glucosyl donor and acceptor substrate in the presence of 80 μg of the enzyme were determined from Lineweaver-Burk plots. The *k*cat was determined by dividing *V*max by the enzyme concentration.

## Authors’ contributions

BF: conception, coordination, design, experiments, data analysis, interpretation and writing of the manuscript; KG, KS, and BF: experiments, data analysis, interpretation and writing of the manuscript; JM, CK: metabolites profiling, isolation and characterization; SC conception, coordination and writing of the manuscript: MS and BF: student supervision, coordination and writing of the manuscript; MD: Bioinformatic analysis; RD: NAPGEN EST and 13 EST libraries. All authors read, commented and approved the manuscript.

## Supplementary Material

Additional file 1 **Phylogenetic consensus tree of 16 partial flax UGT cDNA depicting 8 clusters as inferred by the UPGMA method using 1000 bootstrap replicates.** Bootstrap values (%) are indicated on the branches. Click here for file

Additional file 2 **Isolation of five full length UGT cDNAs using 5′and 3′ RACE PCR. A**, Amplicons of the 5′ cDNA ends by RACE PCR; **B**, Amplicons of the 3′ cDNA ends by RACE PCR; **C**, Amplicons of the full length UGT cDNAs. M, 1 Kb Plus DNA ladder (Invitrogen, ON, Canada). Click here for file

Additional file 3 **ClustalW multiple amino acid sequence alignment of five flax UGTs.** Consensus amino acids, conservation and quality of conservation are shown. The PSPG motif is boxed in red and the HCGW tetra amino acid residues within the PSPG motif are underlined. Click here for file

Additional file 4 EST abundance of five flax full length UGT cDNAs in 13 tissue-specific EST libraries. Click here for file

Additional file 5 **Multiple sequence alignment of the nucleotide sequences for *UGT74S1 *cDNA (from this study), the genomic sequence of *TrUGT74S1 *(JN088324.1, **[27]**), and g6781 the genomic region corresponding to *UGT74S1 *cDNA in the flax genomic database (http://linum.ca; **[26]**).** The two-headed red arrow indicates the 150 bp missing at the 5′ region of *TrUGT74S1*. The two-headed blue arrow indicates the 104 bp present at the 5′ region of *UGT74S1* but absent from the other two UGTs. The two-headed green arrow indicates the position of the intron. The two-headed pink arrow indicates the 3′ untranslated region. Click here for file

Additional file 6 **Comparative UPLC chromatograms of UGT74S1 and TrUGT74S1 (JN088324.1, **[27]**) showing absence of SDG and SMG peaks in TrUGT74S1 reaction products.** A, enzyme reaction including reaction buffer, SECO, UDP-glucose, and 80 μg of His tag-purified UGT74S1 enzyme. B, enzyme reaction including reaction buffer, SECO, UDP-glucose, and 80 μg of His tag-purified TrUGT74S1 enzyme. Peaks 1, 2, and 3 refer to the SDG, SMG and SECO peaks, respectively. Click here for file

Additional file 7 **Morphological changes of flax seed at different days after anthesis (DAA).** Changes in size, shape and color are shown. 8 days after anthesis (DAA), the seeds are usually white/green, flat and soft; 16 DAA, the seeds are greenish, flat to ovoid, soft to slightly hard; 24 DAA, the seeds are green to yellow, flat to ovoid, slightly soft to hard; 32 DAA, the seeds are usually yellow to brown, flat to ovoid, hard; mature seeds (60 DAA), usually brown or yellow, flat to ovoid, dry and hard. Click here for file

Additional file 8 List of gene specific and degenerated primers used for generating partial UGT sequences. Click here for file

Additional file 9 List of gene specific primers (GSP) used for 5′ and 3′ RACE amplification reactions of UGTs. Click here for file

Additional file 10 List of gene specific primers carrying restriction sites used for expression cloning of the full length UGTs in yeast. Click here for file

Additional file 11 List of gene specific primers for UGTs, PLR, and rRNA used in real time PCR reactions. Click here for file
