# Supplementary figures and images for: Identification and functional characterization of a flax UDP-glycosyltransferase glucosylating secoisolariciresinol (SECO) into secoisolariciresinol monoglucoside (SMG) and diglucoside (SDG)
Source: BMC Plant Biol. 2014 Mar 28;14:82. doi: 10.1186/1471-2229-14-82 (PMC3986616; doi:10.1186/1471-2229-14-82)

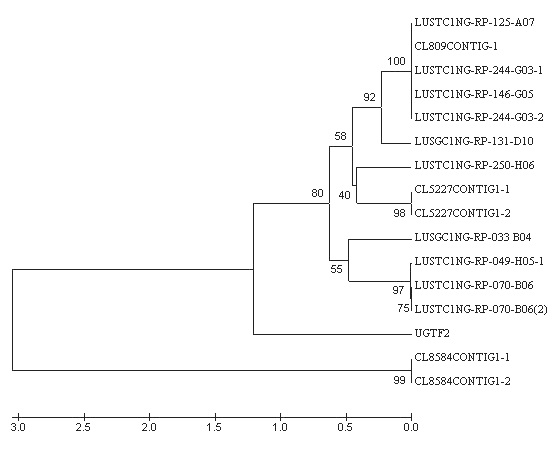

Supplement: Additional file 1 — Phylogenetic consensus tree of 16 partial flax UGT cDNA depicting 8 clusters as inferred by the UPGMA method using 1000 bootstrap replicates. Bootstrap values (%) are indicated on the branches. [file 1471-2229-14-82-S1.tiff]

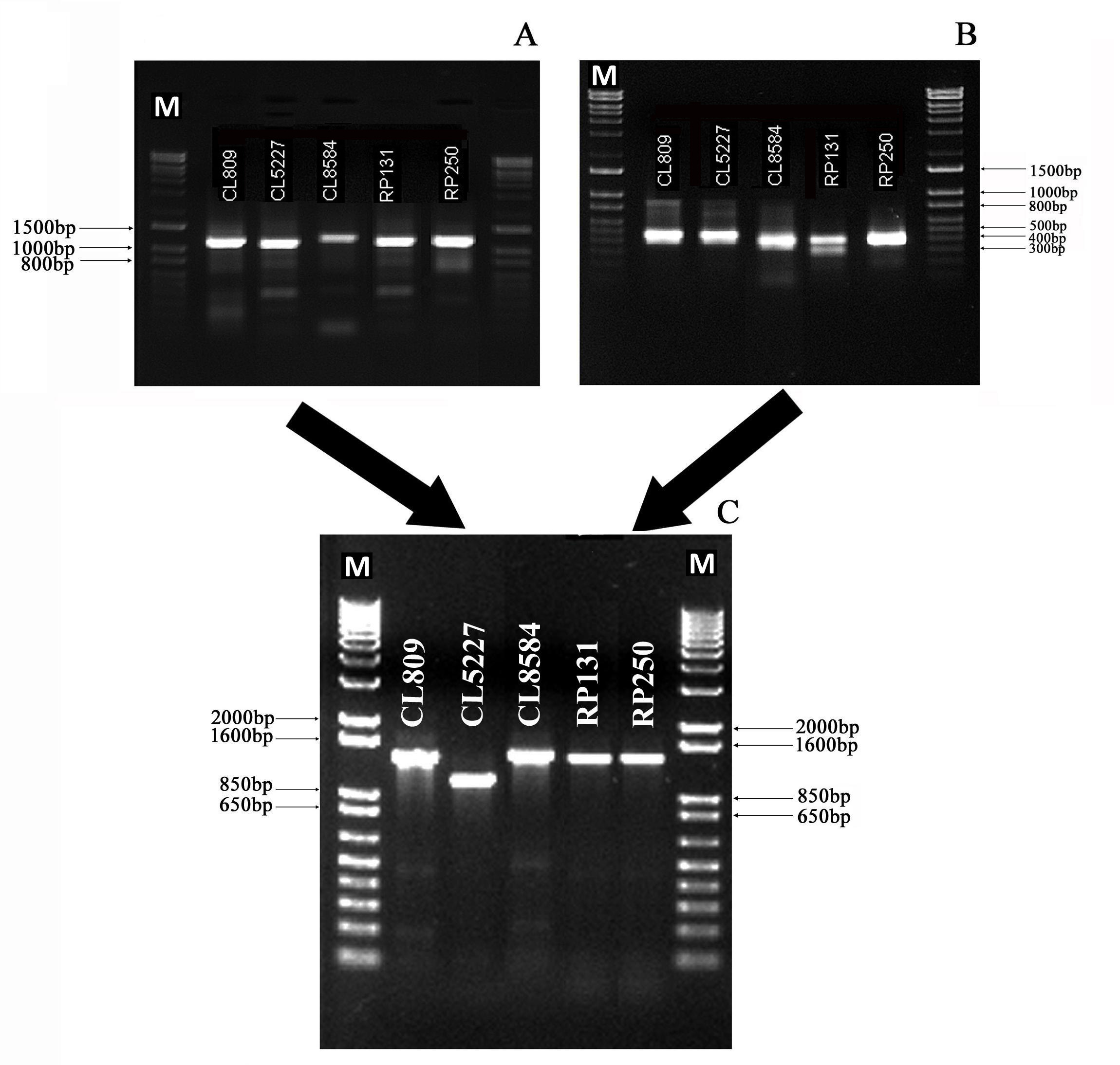

Supplement: Additional file 2 — Isolation of five full length UGT cDNAs using 5′and 3′ RACE PCR. A, Amplicons of the 5′ cDNA ends by RACE PCR; B, Amplicons of the 3′ cDNA ends by RACE PCR; C, Amplicons of the full length UGT cDNAs. M, 1 Kb Plus DNA ladder (Invitrogen, ON, Canada). [file 1471-2229-14-82-S2.tiff]

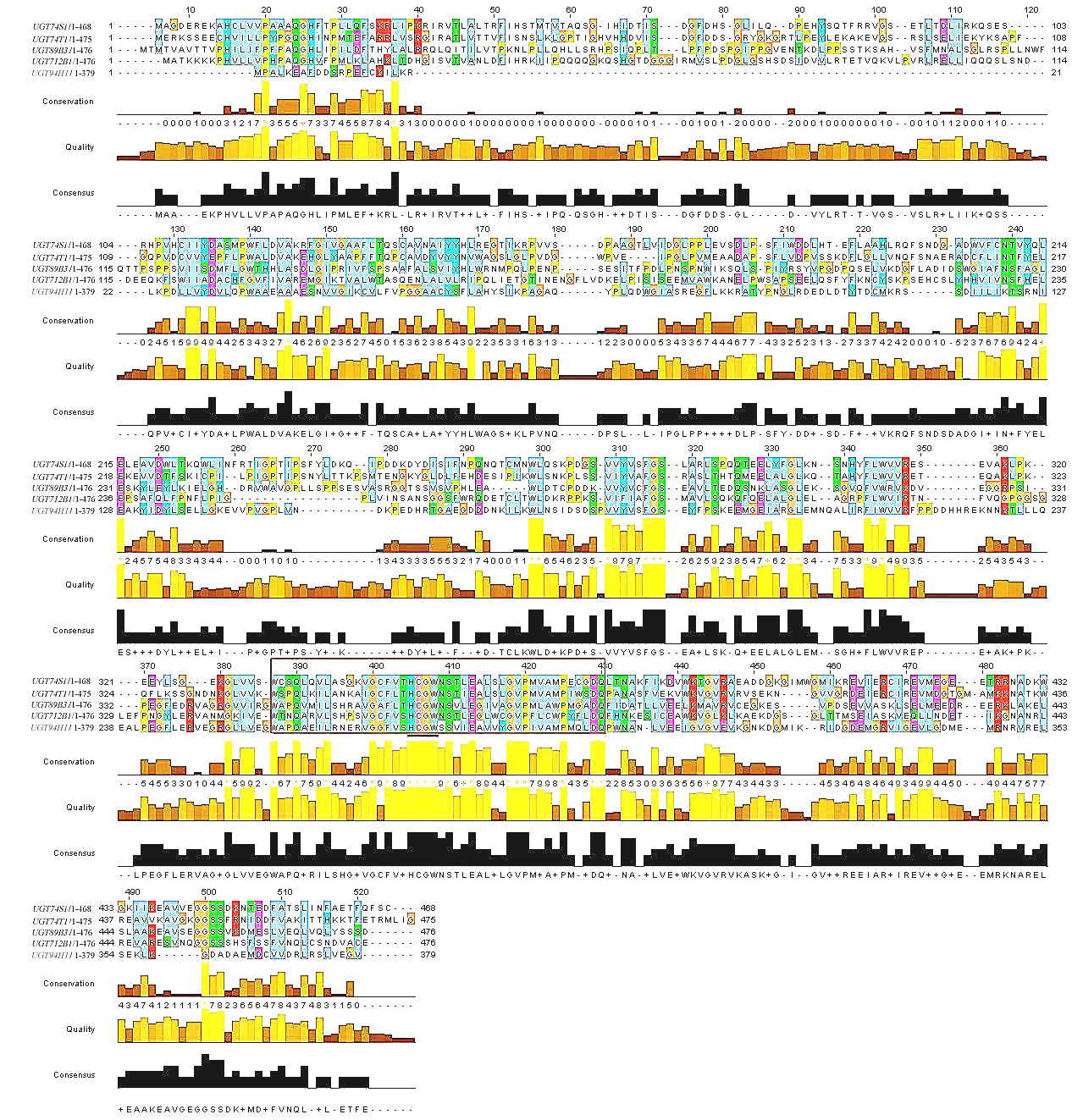

Supplement: Additional file 3 — ClustalW multiple amino acid sequence alignment of five flax UGTs. Consensus amino acids, conservation and quality of conservation are shown. The PSPG motif is boxed in red and the HCGW tetra amino acid residues within the PSPG motif are underlined. [file 1471-2229-14-82-S3.tiff]

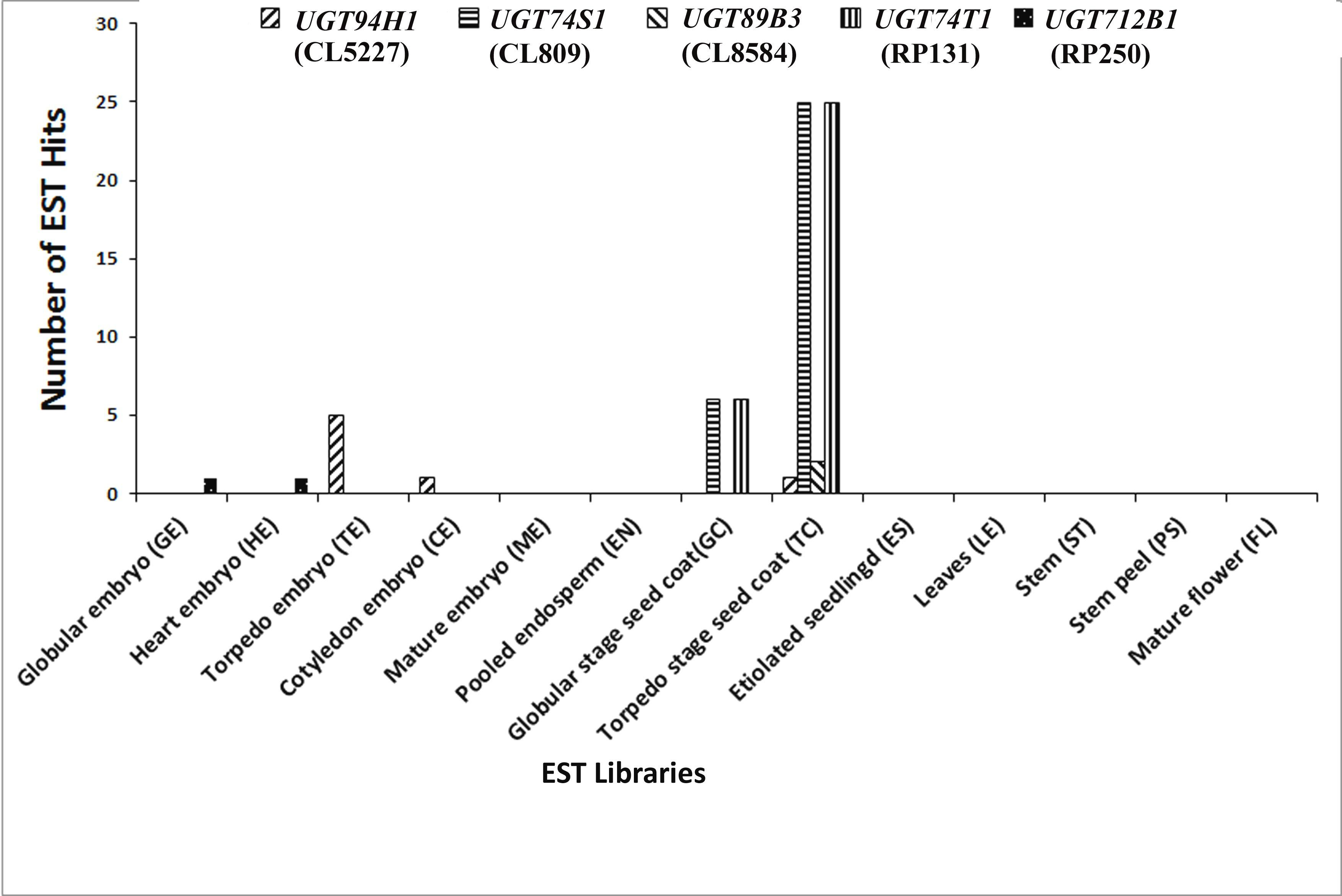

Supplement: Additional file 4 — EST abundance of five flax full length UGT cDNAs in 13 tissue-specific EST libraries. [file 1471-2229-14-82-S4.jpeg]

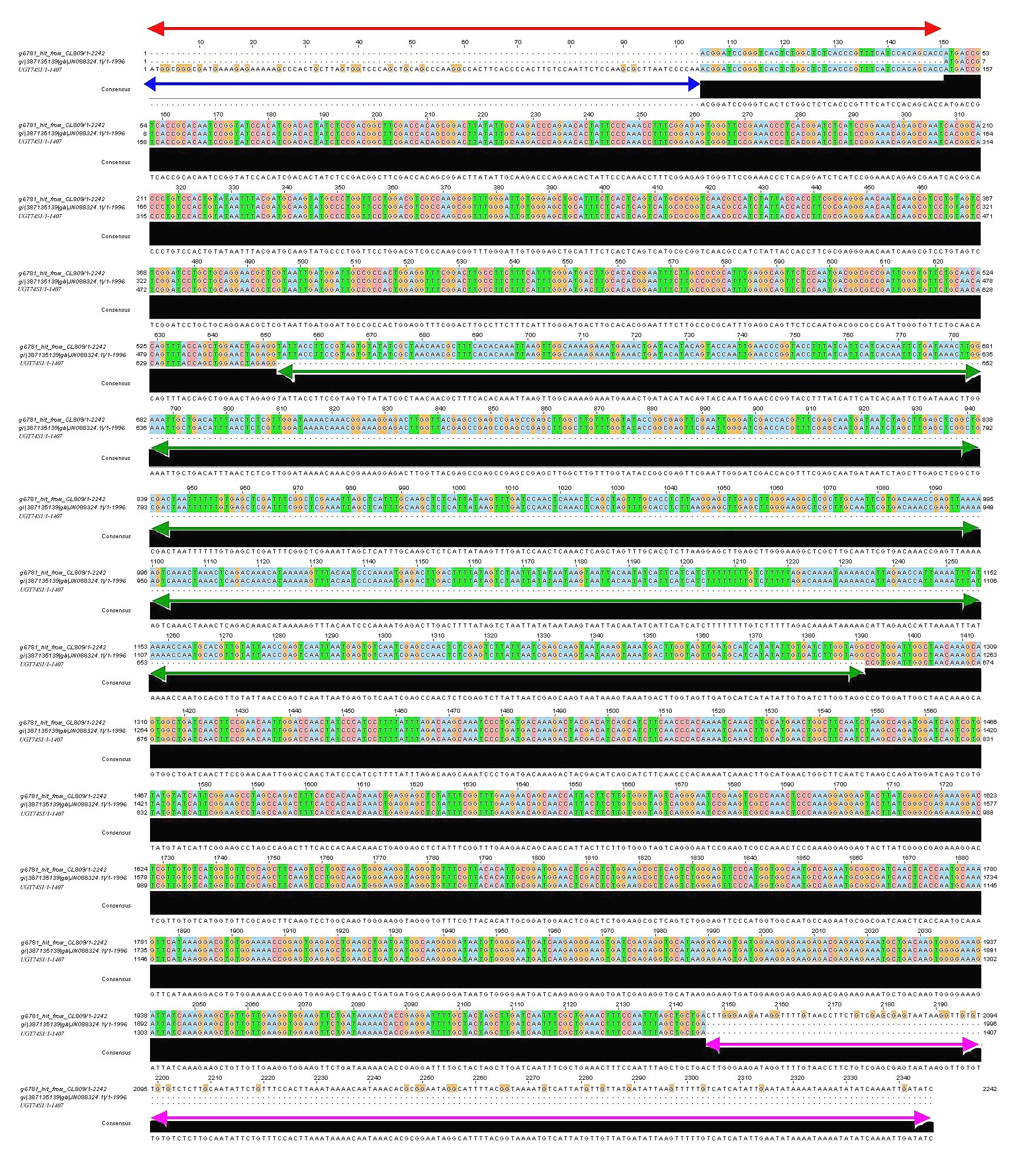

Supplement: Additional file 5 — Multiple sequence alignment of the nucleotide sequences for UGT74S1 cDNA (from this study), the genomic sequence of TrUGT74S1 (JN088324.1, [27]), and g6781 the genomic region corresponding to UGT74S1 cDNA in the flax genomic database (http://linum.ca; [26]). The two-headed red arrow indicates the 150 bp missing at the 5′ region of TrUGT74S1. The two-headed blue arrow indicates the 104 bp present at the 5′ region of UGT74S1 but absent from the other two UGTs. The two-headed green arrow indicates the position of the intron. The two-headed pink arrow indicates the 3′ untranslated region. [file 1471-2229-14-82-S5.tiff]

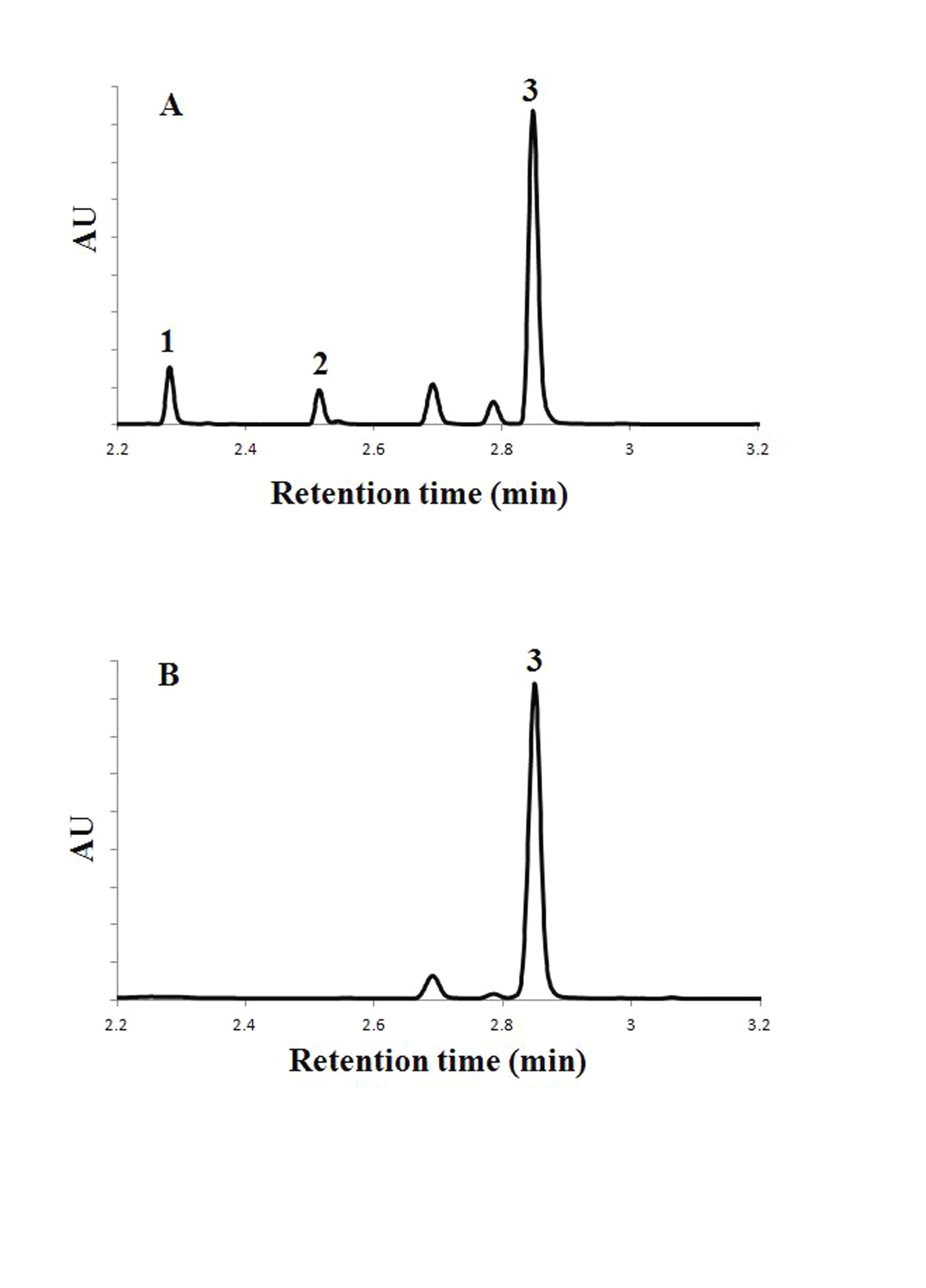

Supplement: Additional file 6 — Comparative UPLC chromatograms of UGT74S1 and TrUGT74S1 (JN088324.1, [27]) showing absence of SDG and SMG peaks in TrUGT74S1 reaction products. A, enzyme reaction including reaction buffer, SECO, UDP-glucose, and 80 μg of His tag-purified UGT74S1 enzyme. B, enzyme reaction including reaction buffer, SECO, UDP-glucose, and 80 μg of His tag-purified TrUGT74S1 enzyme. Peaks 1, 2, and 3 refer to the SDG, SMG and SECO peaks, respectively. [file 1471-2229-14-82-S6.tiff]

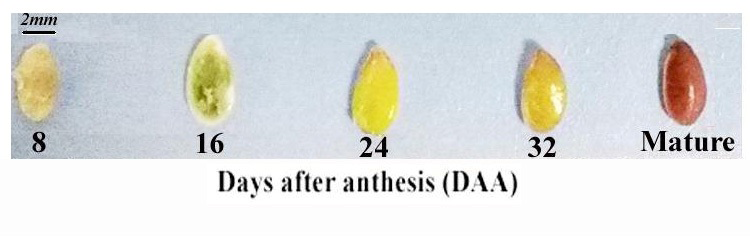

Supplement: Additional file 7 — Morphological changes of flax seed at different days after anthesis (DAA). Changes in size, shape and color are shown. 8 days after anthesis (DAA), the seeds are usually white/green, flat and soft; 16 DAA, the seeds are greenish, flat to ovoid, soft to slightly hard; 24 DAA, the seeds are green to yellow, flat to ovoid, slightly soft to hard; 32 DAA, the seeds are usually yellow to brown, flat to ovoid, hard; mature seeds (60 DAA), usually brown or yellow, flat to ovoid, dry and hard. [file 1471-2229-14-82-S7.tiff]
