# Supplementary material for: Identification and functional characterization of a flax UDP-glycosyltransferase glucosylating secoisolariciresinol (SECO) into secoisolariciresinol monoglucoside (SMG) and diglucoside (SDG)
Source: BMC Plant Biol. 2014 Mar 28;14:82. doi: 10.1186/1471-2229-14-82 (PMC3986616; doi:10.1186/1471-2229-14-82)
Supplement: Additional file 8 — List of gene specific and degenerated primers used for generating partial UGT sequences. [file 1471-2229-14-82-S8.docx]

**Additional file 8 – List of gene specific primers used for generating partial UGT sequences**

| # | **Primer name** | **Forward primers** | **Tm*** | **Reverse primers** | **Tm*** |
| --- | --- | --- | --- | --- | --- |
| 1 | LUSTC1NG-RP-050_B06 | CAACTTCCCTCAACCTATTTGC | 60 | TTCCTCTGGGTGATAAGAACGA | 61 |
| 2 | LUSTC1NG-RP-233_G08 | GCTCCACCAACGACTCCATA | 61 | GTTCCTGACTCACTGCGGTT | 60 |
| 3 | LUSTC1NG-RP-250-H06 | GCAACTTCCCTCAACCTATTTG | 60 | TTCCTCTGGGTGATAAGAACGA | 61 |
| 4 | LUSTC1NG-RP-070-B06 | AGCATTCAGGGCATCTTGAG | 60 | CATTCATCTGGGCAATCCA | 60 |
| 5 | LUSTC1NG-RP-070-B06 | CATTCAGGGCATCTTGAGAACT | 60 | GTCATTCATCTGGGCAATCC | 60 |
| 6 | LUBE1NG-RP-136 | ATTCGTCCCACCTCTTCGCT | 63 | AGGCGTTGAGCCACTGTTCA | 64 |
| 7 | CL5227CONTIG1-1 | ACACAATCCATCTCAGCATCAG | 60 | CATCTGGGTGGTCAGGTTTC | 60 |
| 8 | CL5227CONTIG1-2 | CCATCTCAGCATCAGCATCAC | 62 | TCAGGTTCATCTGGGTGGTC | 61 |
| 9 | CL8584CONTIG1-1 | CGGTAGGATTAGGAGGGACACA | 62 | CCCGCAGGTGATGATACTGAGTCA | 62 |
| 10 | CL8584CONTIG1-2 | GGATTAGGAGGGACACAATGG | 61 | GGGAAGAGGAGTGGTGATAAGG | 61 |
| 11 | LUSGC1NG-RP-033_B04 | GATTCTTCCAGAGGTTCGCC | 61 | CCACCAGGTAATGATGTCGTTC | 61 |
| 12 | LUSGC1NG-RP-131-D10 | GAAACGAAGACCCGCCTTT | 61 | TGGAAGAACTTGCCTTGGG | 61 |
| 13 | LUSTC1NG-RP-125-A07 | AACTTCCACCTTCAACAACAGC | 60 | ACGACATCAGCATCTTCAACC | 60 |
| 14 | LUSTC1NG-RP-244-G03-1 | CGAAACCTATCTTCCCAAGTCA | 60 | CACCACAACAAACTGAGGAGC | 60 |
| 15 | LUSTC1NG-RP-244-G03-2 | AACTTCCACCTTCAACAACAGC | 61 | GCCAGACTTTCACCACAACAA | 61 |
| 16 | LUSTC1NG-RP-146-G05 | CGCAAGAGACACAACACAACC | 62 | GCCAGACTTTCACCACAACAA | 61 |
| 17 | LUSTC1NG-RP-049-H05-1 | AAAGCATTCAGGGCATCTTG | 60 | GTCATTCATCTGGGCAATCC | 60 |
| 18 | LUSTC1NG-RP-049-H05-2 | CATTCAGGGCATCTTGAGAACT | 60 | TTCATCTGGGCAATCCAACT | 60 |
| 19 | CL809CONTIG-1 | AACTTCCACCTTCAACAACAGC | 60 | ACGACATCAGCATCTTCAACC | 60 |
| 20 | 150/605 UGT-F2/R2 | TGGTCTACATCGCCAAACT | 56 | CATCCTCATAATCATCCGC | 56 |

***Tm, melting temperature expressed in degree centigrade**
