# Supplementary material for: Identification and functional characterization of a flax UDP-glycosyltransferase glucosylating secoisolariciresinol (SECO) into secoisolariciresinol monoglucoside (SMG) and diglucoside (SDG)
Source: BMC Plant Biol. 2014 Mar 28;14:82. doi: 10.1186/1471-2229-14-82 (PMC3986616; doi:10.1186/1471-2229-14-82)
Supplement: Additional file 9 — List of gene specific primers (GSP) used for 5′ and 3′ RACE amplification reactions of UGTs. [file 1471-2229-14-82-S9.docx]

**Additional file 9 – List of gene specific primers (GSP) used for 5’ and 3’ RACE amplification reactions of UGTs**

| **Primer name** | **Forward primers** | **Tm*** | **Reverse primers** | **Tm*** |
| --- | --- | --- | --- | --- |
| **CL809 -N** | GCGGCGATCAACTCACCAATGCAAAGTT | 74 | CGCATTCTGGCATTGCCACCATGGGAACT | 78 |
| **GSP** | GCAATGCCAGAATGCGGCGATCAAC | 74 | TGCATTGGTGAGTTGATCGCCGCATT | 74 |
| **CL5227-N** | GGAGCTCGGTGATCGAGGCCGTTGT | 74 | CCCTCCTACCCTCTCGTTCCTCAGAAT | 68 |
| **GSP** | GGTTTGTGAGCCACTGCGGGTGGAG | 74 | CCCGCAGTGGCTCACAAACCCTCCTA | 74 |
| **CL8584-N** | GTTTATTGATGCGACGCTGCTGGT | 67 | GCATCAATAAACTGATCCGCCCCCAT | 71 |
| **GSP** | GGCCGATGGGGGCGGATCAGTTTAT | 74 | CCTCCACCAGCAGCGTCGCATCAATA | 74 |
| **RP-131-N** | GGCGATGAAGAAGAATGCTACCAAA | 65 | CACTGCAACCATGGGAACTCCCAAACT | 71 |
| **GSP** | ATGGATGGGACTGGGATGGCGATGA | 73 | GTCGCTCCATATGGGCACTGCAACCA | 74 |
| **RP-250-N** | GTGGAGTTCCGTTTCTGTGCTGGCCTTA | 72 | CCTCCAGGGTGGAGTTCCATCCGCAAT | 75 |
| **GSP** | CCACCCTGGAAGGGTTGTGGTGTGG | 73 | CGGAACTCCACACCACAACCCTTCCA | 73 |
| **RP-033-N** | GATCAGAAGATGAATGCAGCGTTGTTGGT | 70 | TCTCCAAACTCGAGTTCCACCCGCAAT | 72 |
| **GSP** | CCTGGCCTCAGCACGCAGATCAGAA | 73 | CCGGCACCCCATTGGTTATGCTCTCCAA | 76 |
| **RP-070 N** | TGGTCCCCTCTGATCGGCCATGCTA | 75 | TCCTTTCGCACTGCGGGTGGAACTC | 75 |
| **GSP** | CCCCGCCTCGAATCTCATCCTCGTC | 75 | CGGCTGGGCGCCTCAACTGCTTATT | 75 |
| **UGTF2 N** | GCCCAGACCCACGTGCATCGTCTCT | 75 | CGGCCTGGAAGATCCCGAGGATTGA | 75 |
| **GSP** | CAGGGCTCCCCGAAAACTGCGAGAA | 75 | CGGGCTGCGTTGAGAGGTGTTGTGA | 75 |

***Tm, melting temperature expressed in degree centigrade; Primer’s name followed by N stands for nested primers**
