# Supplementary material for: Identification and functional characterization of a flax UDP-glycosyltransferase glucosylating secoisolariciresinol (SECO) into secoisolariciresinol monoglucoside (SMG) and diglucoside (SDG)
Source: BMC Plant Biol. 2014 Mar 28;14:82. doi: 10.1186/1471-2229-14-82 (PMC3986616; doi:10.1186/1471-2229-14-82)
Supplement: Additional file 10 — List of gene specific primers carrying restriction sites used for expression cloning of the full length UGTs in yeast. [file 1471-2229-14-82-S10.docx]

**Additional file 10 – List of gene specific primers carrying restriction sites used for expression cloning of the full length UGTs in yeast**

|  | **Forward Primers** | | **Reverse Primers** | |
| --- | --- | --- | --- | --- |
| **UGTs** | **RE** | **Primer sequence** | **RE** | **Primer sequence** |
| CL809 | EcoRI | AAAGAATTCATGGCGGGCGATGAAAG | XhoI | TTTCTCGAGACTCGCTCGACAGAAGGTTACA |
| CL5227 | BamHI | AAAGGATCCATGCCCGCTTTGAAGG | XhoI | TTTCTCGAGATCATTGATTTTGGCCCTCAC |
| CL8584 | BamHI | AAAGGATCCATGACCATGACGGTGGCCG | NotI | TTTGCGGCCGCAACGGTCTCATGCATTAACAT |
| RP131 | BamHI | AAAGGATCCATGGAAAGAAAATCTAGC | XbaI | TTTTCTAGACGGAGATCGAGTAACACAGACC |
| RP-250 | BamHI | AAAGGATCCATCATGGCGACCAAGAAG | EcoRI | TTTGAATTCTTATTGGACTAAAACCAATCATCA |
| JN088324.1 | EcoRI | AAAGAATTCAGCACCATGACCGTCAC | XhoI | TTTCTCGAGACTCGCTCGACAGAAGGTTACA |

**RE, restriction enzyme; Restriction sites are underlined.**
