# Supplementary material for: Identification and functional characterization of a flax UDP-glycosyltransferase glucosylating secoisolariciresinol (SECO) into secoisolariciresinol monoglucoside (SMG) and diglucoside (SDG)
Source: BMC Plant Biol. 2014 Mar 28;14:82. doi: 10.1186/1471-2229-14-82 (PMC3986616; doi:10.1186/1471-2229-14-82)
Supplement: Additional file 11 — List of gene specific primers for UGTs, PLR, and rRNA used in real time PCR reactions. [file 1471-2229-14-82-S11.docx]

**Additional file 11 – List of gene specific primers for UGTs, PLR, and rRNA used in real time PCR reactions**

| # | **Primer names** | **Forward primers** | **Reverse primers** |
| --- | --- | --- | --- |
| 1 | CL809 | GGATGGAACTCGACTCTGGA | CTTTCCCCACTTGTCAGCAT |
| 2 | CL5227 | GGAGGGTTTGTGAGCCACTG | TCCCCATCTCATCTCCATCA |
| 3 | CL8584 | TTCCGGAAGGATTCGAGGAT | GCAGACTCTCACCGCCATCT |
| 4 | RP131 | TTTGGGAGTTCCCATGGTTG | GCCTCCCTCCATTTGGTAGC |
| 5 | RP250 | ATTTCGTGCAAGGACCAGGA | GCCAGCACAGAAACGGAACT |
| 6 | PLR | TGGCAACGTCAAAGTGGTGT | ACCTGCTCCGCTACGTCCTT |
| 7 | rRNA | ATTCGGCCCGTCTTGAAACA | GGGCCTCCACCAGAGTTTCC |
